# Nucleosomal dsDNA Stimulates APOL1 Expression in Human Cultured Podocytes by Activating the cGAS/IFI16-STING Signaling Pathway

**DOI:** 10.1038/s41598-019-51998-w

**Published:** 2019-10-29

**Authors:** Shamara E. Davis, Atanu K. Khatua, Waldemar Popik

**Affiliations:** 1 Meharry Medical College, Center for AIDS Health Disparities Research, Department of Microbiology and Immunology, Nashville, TN 37208 USA; 2 Department of Internal Medicine, 1005 D. B. Todd Blvd, Nashville, TN 37208 USA

**Keywords:** Cell biology, Drug discovery

## Abstract

APOL1 alleles G1 and G2 are associated with faster progression to lupus nephritis (LN)-associated end-stage renal disease (LN-ESRD) in African Americans. Increased levels of type I interferons (IFNs) and nucleosome-associated double-stranded DNA (dsDNA) fragments (nsDNA) are the hallmark of this disease. Here, we identify cyclic GMP-AMP synthase (cGAS) and interferon-inducible protein 16 (IFI16) as the major DNA sensors in human immortalized podocytes. We also show that nsDNA triggers the expression of APOL1 and IFNβ via IRF3 activation through the cGAS/IFI16-STING pathway. We demonstrate that maximal APOL1 expression also requires the activation of type I IFN receptor (IFNAR) and STAT1 signaling triggered by IFNβ produced in response to nsDNA, or by exogenous IFNβ. Finally, we show that STAT1 activation is sufficient to upregulate IFI16, subsequently boosting APOL1 expression through a positive feedback mechanism. Collectively, we find that nsDNA-induced APOL1 expression is mediated by both IFNβ-independent and dependent signaling pathways triggered by activation of the cGAS/IFI16-STING pathway. We propose that simultaneous inhibition of STING and the IFNAR-STAT1 pathway may attenuate IFI16 expression, reduce IFI16-cGAS cross-talk, and prevent excessive APOL1 expression in human podocytes in response to nsDNA.

## Introduction

Chronic kidney disease (CKD) affects approximately 30 million people in the US. African Americans, representing ~13% of the US population, account for ~32% CKD cases in the US^1,2^. They are also four times more likely to develop end-stage renal disease (ESRD) than Americans of European ancestry. It has been shown that two coding variants of the apolipoprotein L1 (APOL1) gene, G1 and G2^3,4^, predispose African Americans to a range of kidney diseases, including human immunodeficiency virus-associated nephropathy (HIVAN), focal segmental glomerulosclerosis (FSGS), hypertension-attributed ESRD, sickle cell nephropathy, and lupus nephritis (LN)-associated ESRD (LN-ESRD)^3–7^.

Injury of kidney glomerular podocytes^8–10^ is one of the hallmarks of LN that develops in ~40–70% of patients with systemic lupus erythematosus (SLE)^11^. Both *in vitro*^12–16^ and *in vivo*^17^ overexpression of APOL1 in podocytes is highly toxic. These observations suggest that faster progression to LN-ESRD in African Americans may be due to overexpression of the APOL1 risk variants in the podocytes within this population. On the other hand, incomplete penetrance of the APOL1 risk alleles in APOL1-associated kidney disease implicates environmental factors as major contributors to APOL1 expression and kidney injury in African Americans^18^. How these factors affect lupus progression is unknown.

The presence of high levels of blood-circulating nucleosome-associated DNA fragments in lupus patients^19,20^ suggests that the activation of DNA-sensing receptors may also account for, at least in part, increased IFN expression^21^. In this regard, the endosomal Toll-like receptor 9 (TLR9)^22^, cytosolic cyclic GMP-AMP synthase (cGAS)^23^, and interferon-inducible protein 16 (IFI16)^24^ have been implicated in SLE^25–29^. However, in contrast to cGAS and IFI16, TLR9 is expressed only in podocytes from patients with active LN^30^. This observation suggests that in normal kidney podocytes, cGAS and/or IFI16 may be the major DNA-sensing receptors that drive the expression of APOL1 and IFN and subsequently promote progression to LN in SLE patients.

Interaction of cytosolic dsDNA with cGAS stimulates synthesis of cyclic GMP-AMP (2′3′ cGAMP), which binds to and induces conformational changes in stimulator of interferon genes (STING), subsequently triggering the translocation of STING from the endoplasmic reticulum (ER) to ER-Golgi intermediate compartments (ERGIC)^31^. In ERGIC, STING recruits TANK binding kinase 1 (TBK1), which phosphorylates STING and interferon regulatory factor 3 (IRF3). Dimers of phosphorylated IRF3 subsequently translocate to the nucleus, where they stimulate transcription of *IFNβ* and IFN-stimulated genes. Whether the dsDNA-sensing, STING-dependent cGAS and IFI16 pathways are functional and promote APOL1 expression in human podocytes has not been evaluated.

Given that IFN responses triggered by cGAS and IFI16 strongly depend on the length of the encountered dsDNA^24,32^, it is possible that both DNA sensors are optimally activated by dsDNA of different sizes and/or structures, as demonstrated for cGAS^33,34^. In this regard, it has been shown that IFI16 binds dsDNA in a length-dependent manner and cooperatively assembles into filaments on the bound dsDNA^35^. Interestingly, IFI16 oligomerization is optimal on dsDNA of ~150 bp in length, which is comparable to the size of nucleosomal DNA. In view of recent reports demonstrating that the cross-talk between cGAS and IFI16 regulates IFNβ expression in human keratinocytes^36^ and human macrophages^37^, it is conceivable that the strength of responses elicited by viral or synthetic DNA may differ from that triggered by nucleosomal dsDNA (nsDNA) detected in lupus patients^19^.

Here, we demonstrate that cGAS and IFI16 are the major DNA-sensing receptors in human immortalized AB8/13 podocytes that trigger the expression of APOL1 and IFNβ in response to cytosolic nsDNA via activation of the cGAS/IFI16-STING pathway. Furthermore, STING activation promotes IRF3 phosphorylation. Phosphorylated IRF3 directly induces the transcription of *APOL1* and *IFNβ*. IFNβ expression activates type I IFN receptor (IFNAR), which, through the IFNAR-associated JAK1 kinase, triggers STAT1 phosphorylation and upregulates expression of APOL1 and IFI16. We show that levels of IFI16 in unstimulated podocytes are significantly lower than those of cGAS, suggesting that increased IFI16 levels in response to IFNβ may enhance the cross-talk with cGAS and result in higher APOL1 expression. However, findings from a previous report^35^ suggest that even low levels of IFI16 may be sufficient to engage nsDNA and contribute to APOL1 expression.

Collectively, we demonstrate that maximal APOL1 expression requires activation of STING-mediated IRF3 signaling as well as IFNAR-induced STAT1 signaling triggered by IFNβ expressed in response to nsDNA, or by exogenous IFNβ. We propose that simultaneous inhibition of STING and the IFNAR-STAT1 pathway may attenuate expression of endogenous IFNβ and IFI16, as well as inhibit signaling mediated by exogenous IFNβ. Together, these events will prevent excessive APOL1 expression in human podocytes.

## Results

### nsDNA stimulates APOL1 expression in human immortalized AB8/13 and MMC111.3 podocytes

**Figure 1:**
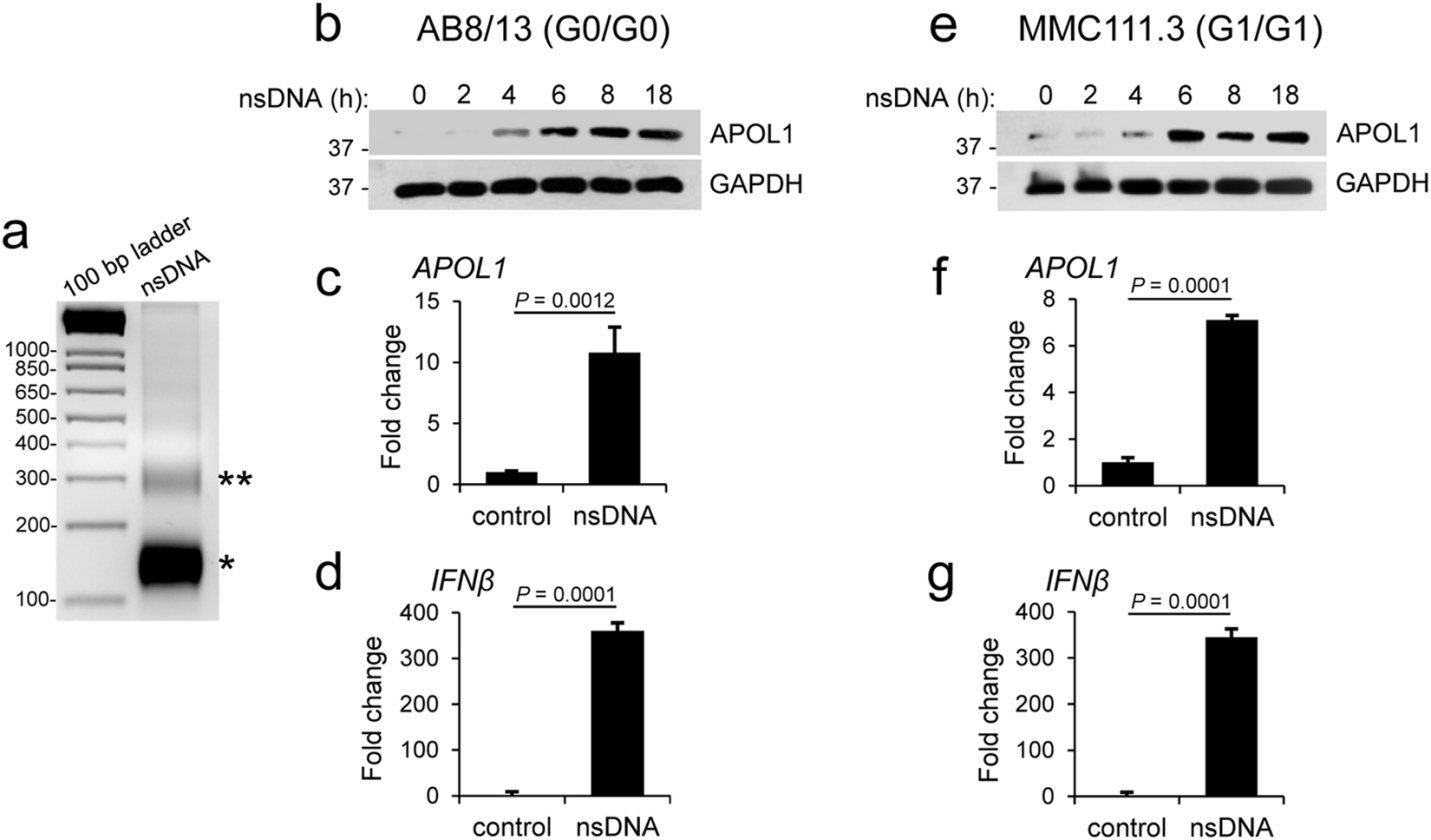
Nucleosome-derived dsDNA (nsDNA) stimulates APOL1 expression in human immortalized AB8/13 and urine-derived MMC111.3 podocytes. (**a**) Analysis of nsDNA prepared from the nuclei of AB8/13 cells on 2% agarose gel (500 ng/lane) followed by ethidium bromide staining shows that over 95% of nsDNA was mono-nucleosomal DNA (approximately 146 bp). Stars indicate mono- (*) and di- (**) nucleosomal DNA. (**b**) APOL1 protein expression in AB8/13 podocytes transfected with 1 μg ml^−1^ nsDNA for the indicated times (h) was analyzed by Western blotting. Protein size markers (kDa) are shown. GAPDH protein levels served as the loading control. The membrane was probed for APOL1 and re-probed for GAPDH. The full images are shown in Supplementary Fig. S1. (**c,d**) Expression of *APOL1* (**c**) and *IFNβ* (**d**) mRNA in mock-transfected AB8/13 cells (control) and cells transfected with 1 μg ml^−1^ nsDNA for 18 h was analyzed by qRT-PCR. (**e**) APOL1 protein expression in MMC111.3 podocytes transfected with 1 μg ml^−1^ nsDNA for the indicated times (h) was analyzed by immunoblotting. The membrane was probed for APOL1 and re-probed for GAPDH. Full images of the blots are shown in Supplementary Fig. S1. (**f**,**g**) Expression of *APOL1* (**f**) and *IFNβ* (**g**) mRNA in mock-transfected MMC111.3 (control) and cells transfected with 1 μg ml^−1^ nsDNA for 18 h was analyzed by qRT-PCR. mRNA expression was normalized to *GAPDH* mRNA levels. Data are expressed as means ± SEM of four (**c**,**d**) or three (**f**,**g**) biological replicates (unpaired Student’s t-test).

Recent studies have demonstrated a strong association between ESRD in African Americans with LN and APOL1 nephropathy risk alleles G1 and G2^7^; however, the mechanism underlying this association remains elusive. The presence of elevated levels of blood-circulating nsDNA in LN patients^19,20,38–40^ prompted us to investigate whether cytosolic accumulation of nsDNA induces APOL1 expression in human immortalized AB8/13 podocytes^41^ that carry the wild-type (G0) *APOL1*. These cells have been widely used to assess the toxic effects of APOL1^12,14–16^. We prepared nsDNA from the nuclei of AB8/13 podocytes using Atlantis, the dsDNA-specific endonuclease that does not degrade dsDNA associated with nucleosomes. We found that mono-nucleosomal DNA fragments made up over 95% of the isolated nsDNA (Fig. 1a). Transfection of nsDNA into AB8/13 podocytes induced APOL1 protein accumulation in a time-dependent manner, and the levels peaked at 8–18 h post transfection (Fig. 1b). Correspondingly, we observed an increase of over 10-fold in *APOL1* mRNA accumulation at 18 h post transfection (Fig. 1c). In agreement with previous findings on dsDNA^24,42,43^, we observed that nsDNA robustly stimulated the expression of *IFNβ* mRNA in AB8/13 podocytes (Fig. 1d).

We have also shown that nsDNA stimulates expression of APOL1 protein and *APOL1* mRNA as well as *IFNβ* mRNA in human urine-derived MMC111.3 podocytes homozygous for *APOL1* G1 (Fig. 1e–g). Together, these results show that nsDNA stimulates expression of both wild-type (G0) and G1 APOL1 to a similar extent in human immortalized AB8/13 and MMC111.3 podocytes, respectively.

### nsDNA-mediated APOL1 expression in human immortalized AB8/13 podocytes involves activation of the STING-TBK1-IRF3 pathway

**Figure 2:**
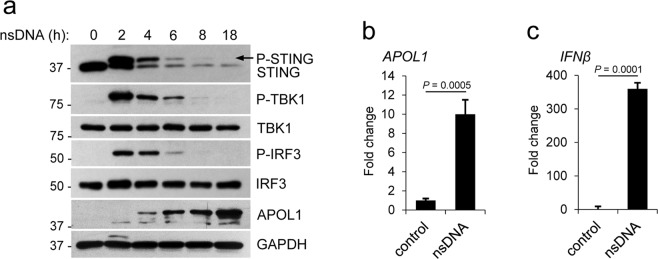
nsDNA-mediated APOL1 expression in human immortalized AB8/13 podocytes involves activation of the STING-TBK1-IRF3 pathway. (**a**) AB8/13 podocytes were transfected with 1 μg ml^−1^ nsDNA for the times indicated. Expression levels of APOL1, STING, TBK1, and IRF3 were analyzed by immunoblotting. STING and phospho-STING (P-STING, marked by an arrow) were detected using antibodies against STING. GAPDH protein levels (loading control) and protein size markers (kDa) are indicated. The blot images were obtained from different gels. The blot probed for IRF3 was re-probed for APOL1. Other blot images were cropped from individually probed blots. Full images of the blots are shown in Supplementary Fig. S2. (**b,c**) Expression of *APOL1* (**b**) and *IFNβ* (**c**) mRNA in mock-transfected AB8/13 cells (control) and cells transfected with 1 μg ml^−1^ nsDNA for 18 h was analyzed by qRT-PCR. mRNA expression was normalized to *GAPDH* mRNA levels. Data are expressed as means ± SEM of four biological replicates (unpaired Student’s t-test).

Cytosolic dsDNA triggers inflammatory responses through the engagement of several recently discovered dsDNA-sensing receptors^42,44^. Among these receptors, cGAS and IFI16 have been implicated in SLE progression^25–28,45^. Since the STING-TBK1-IRF3 signaling pathway plays a central role in sensing cytosolic nucleic acids^46^, we tested if activation of this pathway coincided with APOL1 expression in human immortalized AB8/13 podocytes transfected with nsDNA. We observed a time-dependent phosphorylation of STING (at serine 366), TBK1 (at serine 172), and IRF3 (at serine 386). Phosphorylation of STING, TBK1 and IRF3 observed at 2 h post transfection, gradually decreased and was undetectable by 18 h (Fig. 2a). APOL1 protein levels gradually increased and peaked at 18 h post transfection, which corresponded to ~10-fold increase in *APOL1* mRNA expression (Fig. 2b). We also observed a robust expression of *IFNβ* mRNA in nsDNA-transfected AB8/13 podocytes (Fig. 2c). Together, these results indicate that the STING-TBK1-IRF3 pathway is functional in AB8/13 podocytes and thus may contribute to the expression of APOL1 and IFNβ in these cells.

### siRNA-mediated knockdown of STING, TBK1, or IRF3 inhibits nsDNA-induced APOL1 expression in human AB8/13 podocytes

**Figure 3:**
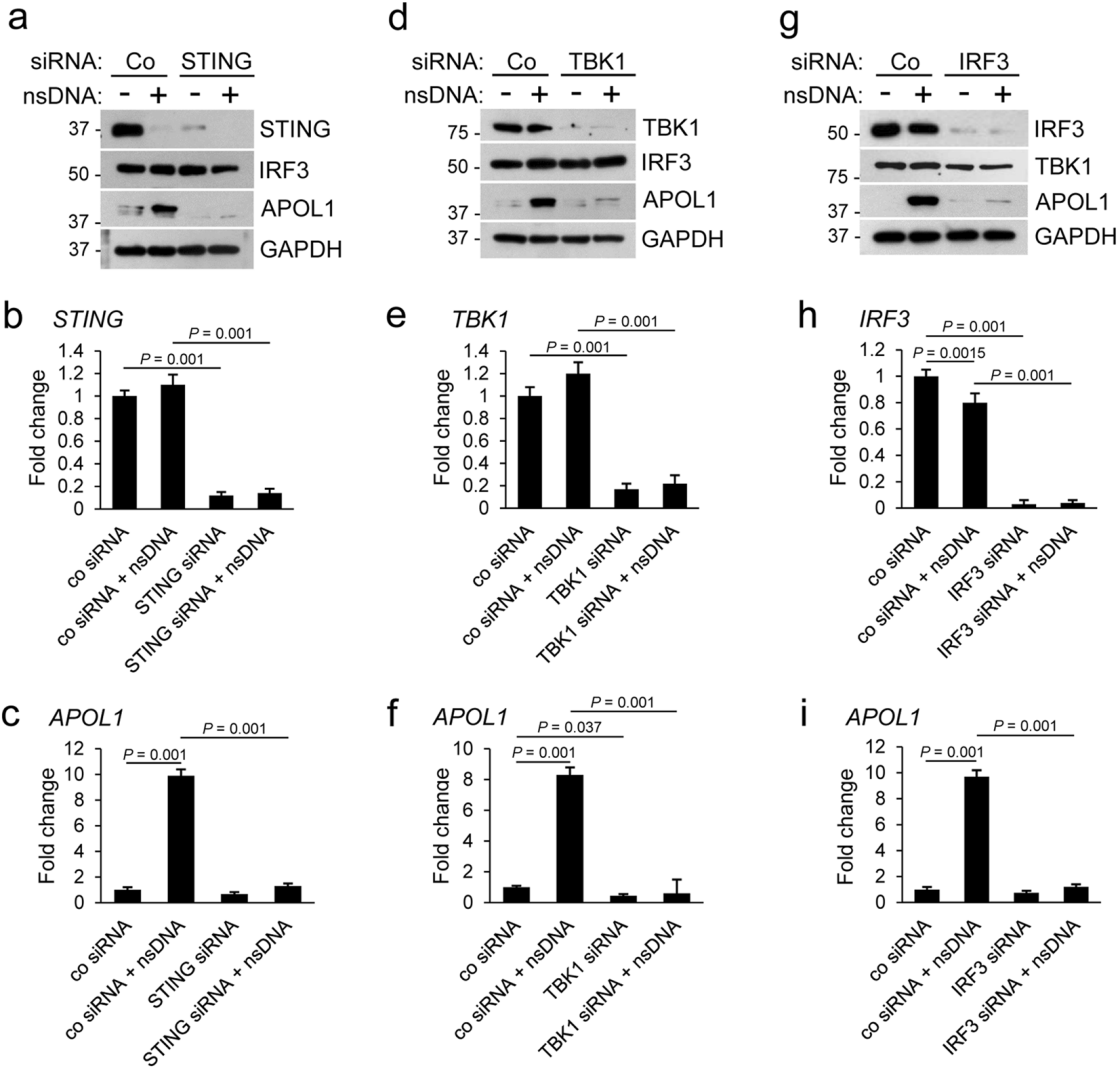
Knockdown of STING, TBK1, or IRF3 inhibits nsDNA-mediated APOL1 expression in human immortalized AB8/13 podocytes. AB8/13 podocytes were transfected with a non-targeting control siRNA (Co) or siRNA pool targeting *STING* (**a**–**c**), *TBK1* (**d**–**f**), or *IRF3* (**g**-**i**) for 48 h, and subsequently transfected with 1 μg ml^−1^ nsDNA for 18 h. Expression of indicated proteins (**a**,**d**,**g**) was analyzed by immunoblotting. Protein size markers (kDa) are shown. The blot images in (**a**) were obtained from different gels. One blot was probed for IRF3 and re-probed for GAPDH. Blot images in (**d**) were obtained from different gels. One blot was probed with TBK1 and re-probed with GAPDH, while two other blots were probed for APOL1 and TBK1. The blot images in (**g**) were obtained from different gels. The blot probed for TBK1 was re-probed for GAPDH. Two other blots were probed individually for IRF3 and APOL1. Full images of the blots are shown in Supplementary Fig. S3. Expression of *STING* (**b**), *TBK1* (**e**), *IRF3* (**h**), and *APOL1* (**c**,**f**,**i**) mRNA was analyzed by qRT-PCR and normalized to *GAPDH* mRNA levels. Data are expressed as means ± SEM from three biological replicates (one-way ANOVA with post-hoc Tukey test).

To confirm that the nsDNA-induced APOL1 expression is mediated by the STING-TBK1-IRF3 pathway, we transiently knockdown the expression of STING, TBK1, and IRF3 using a standard siRNA approach. As assessed by western blotting, transfection of a pool of target-specific siRNAs reduced expression of STING, TBK1, and IRF3 by >90% after 48 h. In cells subsequently transfected with nsDNA for 18 h, knockdown of STING (Fig. 3a), TBK1 (Fig. 3d), or IRF3 (Fig. 3g) strongly inhibited APOL1 expression, as compared to cells transfected with a non-targeting control siRNA. qRT-PCR analysis confirmed 85–95% reduction in *STING*, *TBK1* and *IRF3* mRNA levels that were only marginally increased in cells transfected with nsDNA (Fig. 3b,e,h). Similarly, expression of *APOL1* mRNA in cells transfected with siRNA pools targeting STING, TBK1, or IRF3 and subsequently transfected with nsDNA was strongly inhibited (Fig. 3c,f,i). Our findings indicate that the transfected nsDNA triggers APOL1 expression in AB8/13 podocytes by engaging the STING-TBK1-IRF3 pathway.

### nsDNA-induced APOL1 expression is not mediated by contaminating cellular RNA

The sensing of dsDNA and pathogenic RNA by cytosolic cGAS and RIG-I-like receptors, respectively, may activate STING^47^ and subsequently induce APOL1 expression. Therefore, we evaluated the possibility that cellular RNA may contaminate our nsDNA preparation and contribute to APOL1 expression mediated by activation of the STING pathway. To do this, we incubated the isolated nsDNA with a mixture of RNase A which specifically hydrolyzes RNA at C and U residues, and RNase T1 which specifically hydrolyzes RNA at G residues. Subsequently, using the RNase A/T1-treated nsDNA and RNA-free synthetic dsDNA90^48,49^, we examined activation of the STING-TBK1-IRF3 pathway and APOL1 expression. The phosphorylation status of STING, TBK1, and IRF3 at 2 h post transfection and the levels of APOL1 protein accumulated at 18 h in response to nsDNA and dsDNA90 were similar (Supplementary Fig. S9), suggesting that RNA was undetectable in our nsDNA preparations and thus did not contribute to APOL1 expression.

### cGAMP stimulates the STING-TBK1-IRF3 pathway and APOL1 expression in AB8/13 podocytes

**Figure 4:**
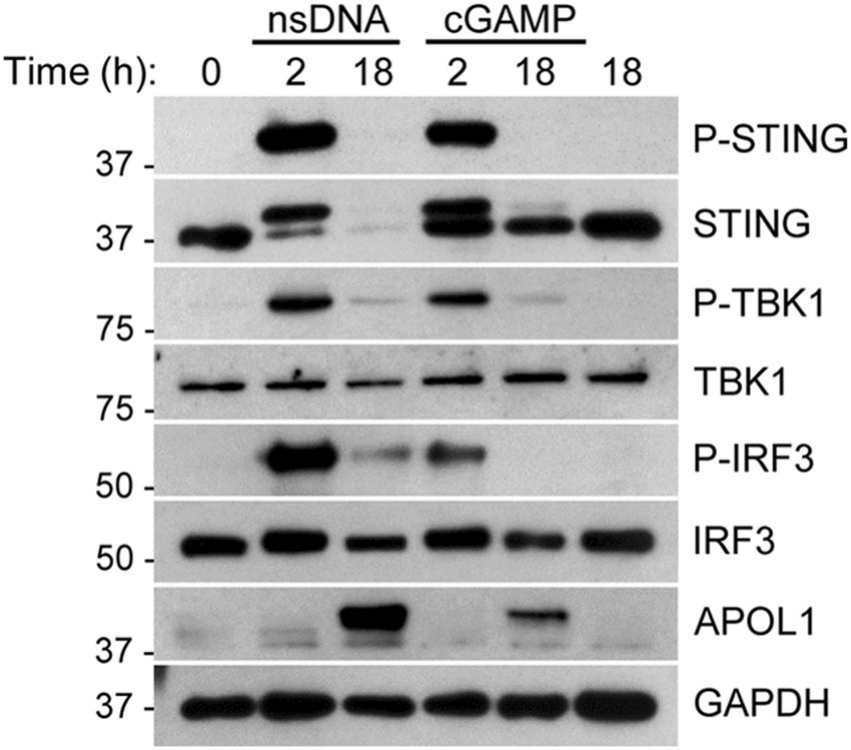
Synthetic cGAMP activates the STING-TBK1-IRF3 pathway and stimulates APOL1 expression. Human immortalized AB8/13 podocytes were transfected with 1 μg ml^−1^ nsDNA or 4 μg ml^−1^ 2′3′-cGAMP for the times indicated. Control cells were either left untreated (0 h) or mock transfected for 18 h. Expression of indicated proteins was analyzed by immunoblotting. Protein size markers (kDa) are indicated. The blot images were obtained from different gels. The blot probed for P-TBK1 was re-probed for STING. The other blots were probed individually for the indicated proteins. Full images of the blots are shown in Supplementary Fig. S4.

Increased expression of cGAS synthase and its product cGAMP has been detected in SLE patients with increased disease activity^25^. Thus, to test whether cGAMP activates the STING-TBK1-IRF3 pathway and stimulates APOL1 expression in AB8/13 podocytes, we transfected the cells with synthetic 2′3′-cGAMP or nsDNA and analyzed the phosphorylation status of STING, TBK1, and IRF3, as well as APOL1 expression (Fig. 4). While both nsDNA and cGAMP induced phosphorylation of STING, TBK1, and IRF3 at 2 h post transfection, cGAMP was less potent than nsDNA. Consequently, nsDNA was a stronger inducer of APOL1 expression than cGAMP.

### Knockdown of cGAS or IFI16 alone is not sufficient to abolish nsDNA-induced APOL1 expression in human AB8/13 podocytes

**Figure 5:**
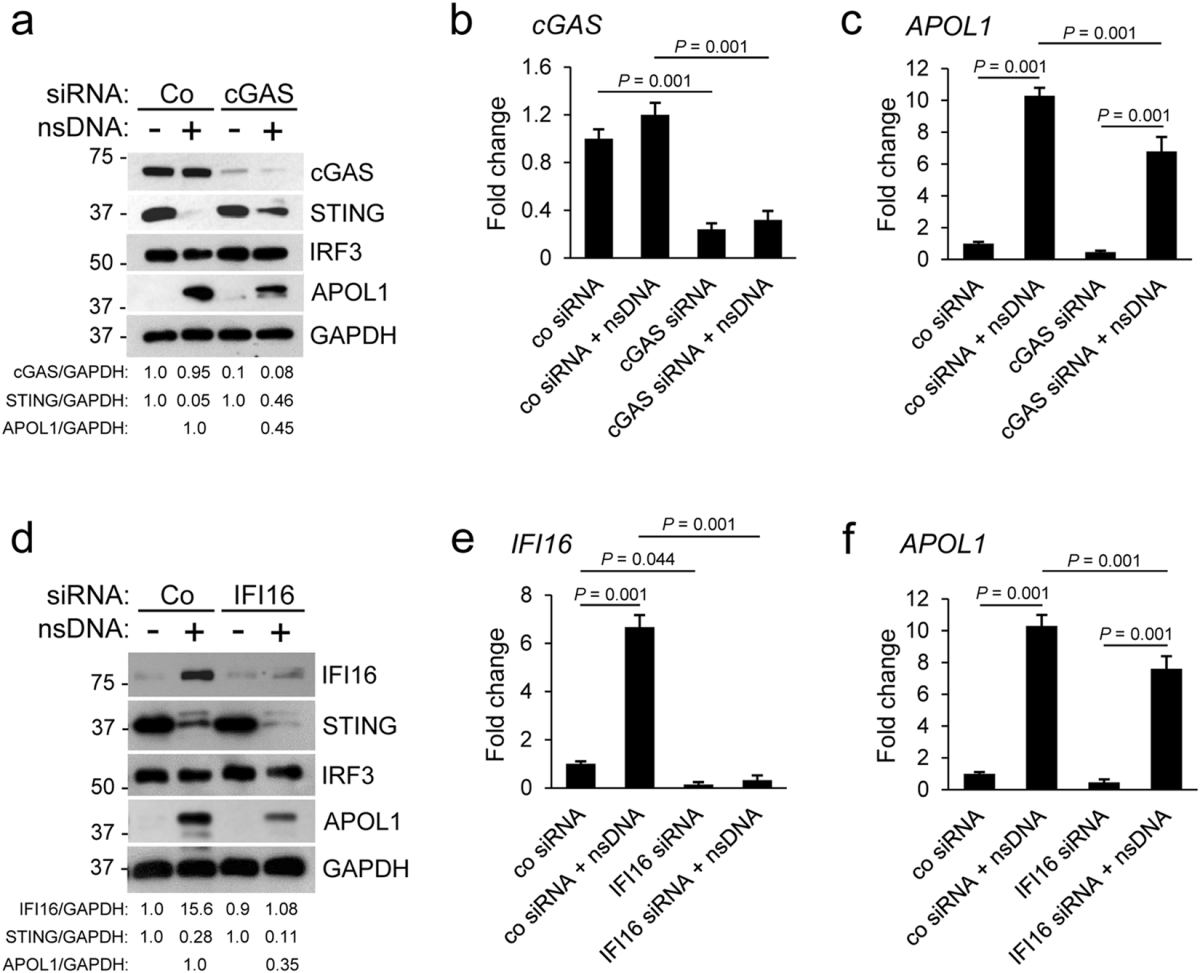
Knockdown of cGAS or IFI16 partly inhibits nsDNA-mediated APOL1 expression in human immortalized AB8/13 podocytes. The cells were transfected for 48 h with control siRNA (Co) or siRNA pool targeting cGAS (**a**–**c**) or IFI16 (**d**–**f**) and subsequently transfected with 1 μg ml^−1^ nsDNA for 18 h. (**a**,**d**) Expression of indicated proteins was analyzed by immunoblotting. Protein size markers (kDa) are shown. Intensities of cGAS, STING, IFI16, APOL1, and GAPDH protein bands were quantified by densitometric scanning. (**a**) Expression levels of cGAS, STING, and APOL1 were normalized against GAPDH levels and presented as cGAS/GAPDH, STING/GAPDH, and APOL1/GAPDH ratios. In cells transfected with control siRNA only, the cGAS/GAPDH and STING/GAPDH ratios were both set as 1.0. The APOL1/GAPDH ratio in cells transfected with control siRNA and nsDNA was set as 1.0. The blot images were obtained from different individually probed gels. (**d**) Expression levels of IFI16, STING, and APOL1 are presented as IFI16/GAPDH, STING/GAPDH, and APOL1/GAPDH ratios. In cells transfected with control siRNA only, the IFI16/GAPDH and STING/GAPDH ratios were both set as 1.0. The APOL1/GAPDH ratio in cells transfected with control siRNA and nsDNA was set as 1.0. The blot images were obtained from different gels. The blot probed for APOL1 was re-probed for IRF3. Full images of the blots in (a) and (d) are shown in Supplementary Fig. S5. Expression of *cGAS* (**b**), *IFI16* (**e**), and *APOL1* (**c**,**f**) mRNA was analyzed by qRT-PCR and normalized to *GAPDH* mRNA levels. Data are expressed as means ± SEM from three biological replicates (one-way ANOVA with post-hoc Tukey test).

To establish the role of cGAS in APOL1 expression in response to nsDNA, we used the siRNA approach to transiently knockdown cGAS in AB8/13 cells. cGAS protein level was reduced by 90% in AB8/13 podocytes transfected with a pool of cGAS-specific siRNAs for 48 h, as compared to cells transfected with non-targeting control siRNA (Fig. 5a), while *cGAS* mRNA levels in these cells decreased by 78% (Fig. 5b). In contrast, the levels of APOL1 protein and *APOL1* mRNA in these cells challenged with nsDNA for 18 h were reduced by 55% and 33%, respectively, as compared to cells transfected with non-targeting control siRNA and challenged with nsDNA (Fig. 5a,c).

Since we ruled out contamination of nsDNA with RNA (Supplementary Fig. S9), we tested the possibility that other cytosolic DNA sensors expressed in AB8/13 podocytes may play a role in nsDNA sensing and inducing APOL1 expression. Increased levels of circulating IFN in lupus patients^50,51^ prompted us to test whether IFN-inducible protein IFI16, identified as a sensor for intracellular DNA^24^ and implicated in SLE progression^28^, plays a role in nsDNA-induced APOL1 expression.

In contrast to the high expression levels of cGAS in unstimulated AB8/13 podocytes, the levels of IFI16 protein and *IFI16* mRNA in unstimulated cells were low but were strongly upregulated in response to nsDNA (Fig. 5d,e). Conversely, in cells transfected with a pool of IFI16-specific siRNAs and subsequently challenged with nsDNA, expression of IFI16 protein and *IFI16* mRNA was reduced by 93% and 95%, respectively (Fig. 5d,e). Comparable to what we observed in cGAS-knockdown AB8/13 podocytes (Fig. 5a–c), depletion of IFI16 by siRNA only partially reduced nsDNA-induced expression of APOL1 protein (by 65%) and *APOL1* mRNA (by 26%) (Fig. 5d,f). These results suggest that both cGAS and IFI16 may be required for maximal APOL1 expression in AB8/13 podocytes.

### cGAS and IFI16 are required for maximal APOL1 expression in human AB8/13 podocytes in response to nsDNA

**Figure 6:**
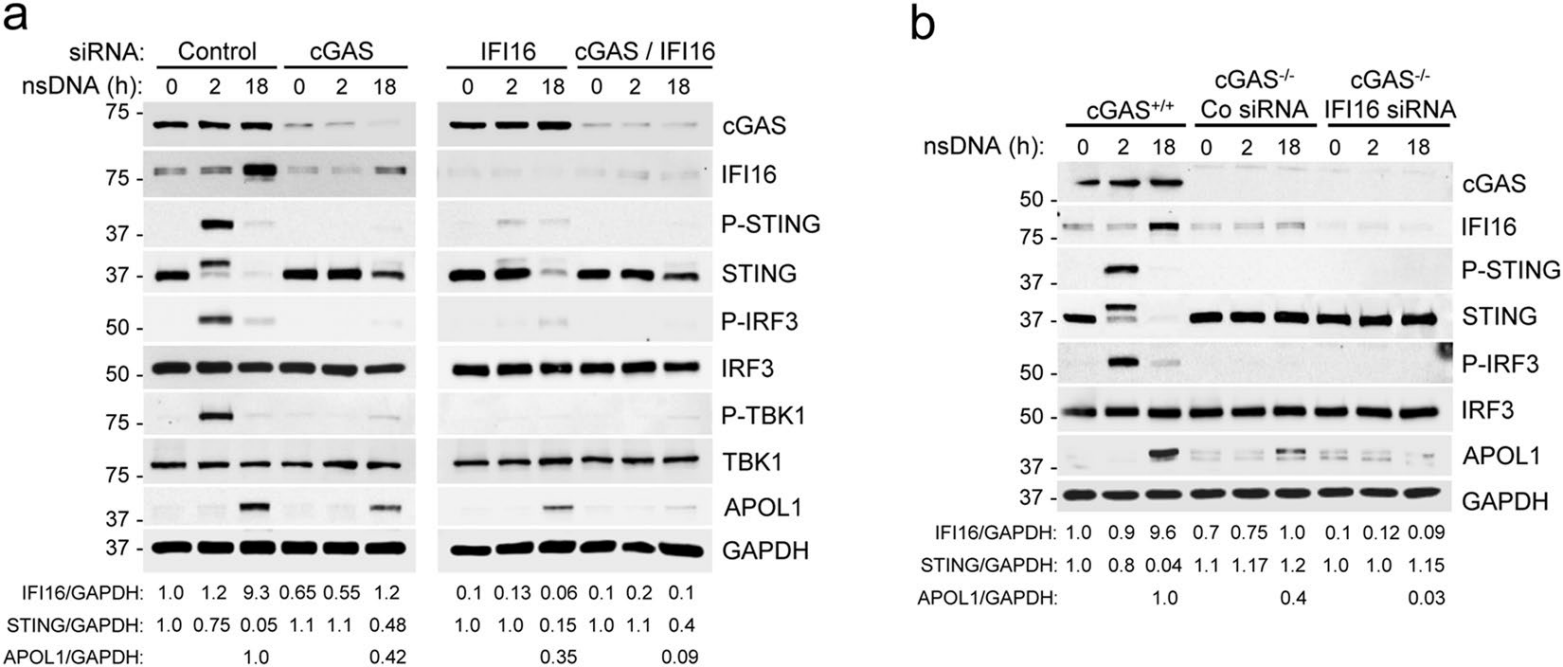
cGAS and IFI16 are required for maximal APOL1 expression in human immortalized AB8/13 podocytes in response to nsDNA. (**a**) AB8/13 podocytes were transfected for 48 h with control siRNA (Co) or siRNA pool targeting either cGAS or IFI16 or both. The cells were subsequently transfected with 1 μg ml^−1^ nsDNA for the times indicated. The blot images were obtained from different gels. Left panel: The blot probed for cGAS was re-probed for GAPDH. The blot probed for APOL1 was re-probed for TBK1. Right panel: The blot probed for cGAS was re-probed for GAPDH. The blot probed for APOL1 was re-probed for TBK1. Expression levels of IFI16, STING, and APOL1 are presented as IFI16/GAPDH, STING/GAPDH, and APOL1/GAPDH ratios. The IFI16/GAPDH and STING/GAPDH ratios in cells transfected with control siRNA only were both set as 1.0. The APOL1/GAPDH ratio in cells transfected with control siRNA and nsDNA (18 h) was set as 1.0. (**b**) cGAS^−/−^ cells generated from parental AB8/13 podocytes (cGAS^+/+^) were transfected for 48 h with control siRNA (Co) or siRNA pool targeting IFI16 and subsequently transfected with 1 μg ml^−1^ nsDNA for the times indicated. Expression of indicated proteins was analyzed by immunoblotting. Protein size markers (kDa) are shown. The blot images were obtained from different gels. The blot probed for cGAS was re-probed for GAPDH. The other blot images were cropped from individually probed blots. The IFI16/GAPDH and STING/GAPDH ratios in cGAS^+/+^ cells exposed to transfection reagent only were both set as 1.0. The APOL1/GAPDH ratio in cGAS^+/+^ cells transfected with nsDNA (18 h) was set as 1.0. Full images of the blots are shown in Supplementary Fig. S6.

To further evaluate the role of cGAS and IFI16 in nsDNA-induced APOL1 expression, we transfected AB8/13 podocytes separately with cGAS siRNA and IFI16 siRNA, or with a combination of cGAS siRNA and IFI16 siRNA (Fig. 6a). Interestingly, cGAS knockdown reduced basal IFI16 expression by 35% and nsDNA-mediated IFI16 induction by 87% (Fig. 6a, left panel), suggesting that IFI16 expression in AB8/13 podocytes is regulated by the cGAS signaling pathway. Accordingly, activation of STING, TBK1, and IRF3, as evaluated by their phosphorylation status, was strongly reduced in cGAS-knockdown cells, as compared to cells transfected with a non-targeting control siRNA. Nonetheless, APOL1 expression was only partly reduced (58%, Fig. 6a, left panel). Unexpectedly, IFI16-knockdown in cells expressing cGAS not only showed lower phosphorylation of STING, TBK1, and IRF3 at 2 h post nsDNA transfection, but also exhibited 85% lower levels of total STING at 18 h after nsDNA challenge (Fig. 6a, right panel). These observations suggest that APOL1 expression may also be regulated by a phosphorylation-independent, STING-mediated pathway. The observation that APOL1 expression was attenuated by 91% in the double-knockdown cells compared to control cells (Fig. 6a, right panel) indicates that cGAS and IFI16 are the major cytosolic nsDNA sensors that mediate APOL1 expression in AB8/13 podocytes.

Since the expression of cGAS protein in AB8/13 podocytes is high and a transient siRNA knockdown of cGAS may be incomplete, we further validated these observations in cGAS^−/−^ knockout AB8/13 podocytes developed using the CRISPR-Cas9 system. The cGAS^−/−^ knockout AB8/13 podocytes showed no detectable cGAS expression, whereas the basal expression of IFI16 were reduced by 30% as compared to cGAS^+/+^ cells (Fig. 6b). Interestingly, the cGAS^−/−^ knockout AB8/13 podocytes express somewhat higher levels of STING than the parental cGAS^+/+^ AB8/13 podocytes (Fig. 6b). Although cGAS is indispensable for nsDNA-mediated induction of IFI16 over the basal level, induction of APOL1 expression was only partially reduced (by 60%) in cGAS^−/−^ cells (Fig. 6b). In this regard, we observed that a transient IFI16 knockdown in cGAS^−/−^ AB8/13 podocytes induced with nsDNA further reduced APOL1 expression by 92%, as compared to nsDNA-challenged cGAS^−/−^ knockout cells transfected with control siRNA (Fig. 6b). Together, these results suggest that cGAS cooperates with IFI16 to fully activate the classical signaling pathway mediated by phosphorylation of STING, TBK1, and IRF3 in response to nsDNA. However, activation of the phosphorylation-independent axis of the STING pathway may also play a role in regulating APOL1 expression in these cells.

### APOL1 expression triggered by nsDNA is mediated by IFNβ-dependent and independent pathways

IFNβ has been shown to promote podocyte injury^52^. However, it is unknown whether cytosolic nsDNA stimulates IFNβ expression in podocytes. We described earlier that nsDNA stimulated a robust expression of *IFNβ* mRNA in AB8/13 podocytes (Figs 1 and 2). To assess the potential contribution of IFNβ induced in response to nsDNA to APOL1 expression, we used Ruxolitinib, a pharmacological inhibitor of JAK1/JAK2 kinases which effectively blocks IFN signaling^53^ and is used in clinical trials for treating lupus^54,55^.

**Figure 7:**
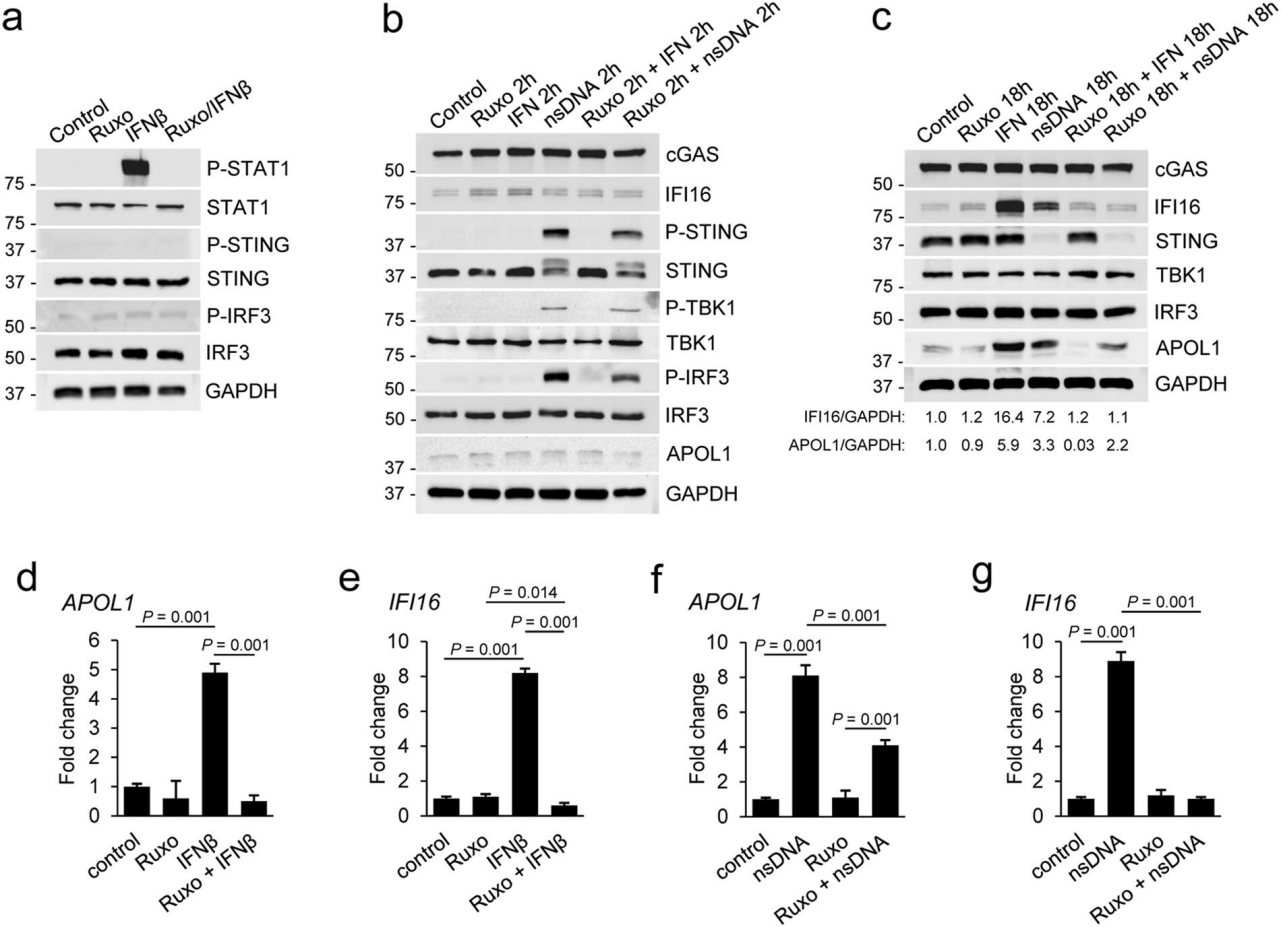
nsDNA-induced APOL1 expression is partly attenuated by JAK1/JAK2 inhibitor Ruxolitinib. (**a**) Ruxolitinib (Ruxo) inhibited IFNβ-mediated phosphorylation of STAT1. Sets of AB8/13 podocytes were treated with DMSO (solvent) only (Control), treated for 2 h with 5 μM Ruxo, treated for 15 min with 10 ng ml^−1^ IFNβ, or pretreated with Ruxo for 2 h followed by IFNβ stimulation for 15 min (Ruxo/IFNβ). The blot images were obtained from different gels. The blot probed for P-STAT1 was re-probed for GAPDH. (**b**,**c**) AB8/13 podocytes were treated with Ruxo and IFNβ as indicated and subsequently transfected with 1 μg ml^−1^ nsDNA for 2 h (**b**) or 18 h (**c**). The IFI16/GAPDH and APOL1/GAPDH ratios in unstimulated cells (Control) were both set as 1.0. The blot images in (**b**) were obtained from different gels. The blot probed for P-IRF3 was re-probed for P-STING. Other blot images were cropped from individually probed blots. The blot images in (**c**) were obtained from different gels. The blot probed for IFI16 was re-probed for cGAS. Other blot images were cropped from individually probed blots. Full images of all blots are shown in Supplementary Fig. S7. (**d**,**e**) Ruxo abolished expression of *APOL1* (**d**) and *IFI16* mRNA (**e**) induced by exogenous IFNβ. (**f**,**g**) Ruxo partially inhibited nsDNA-induced *APOL1* mRNA expression (**f**) but abolished nsDNA-induced *IFI16* mRNA expression (**g**). Expression of *APOL1* and *IFNβ* mRNA was analyzed by qRT-PCR 18 h after transfection with 1 μg ml^−1^ nsDNA. mRNA expression was normalized to *GAPDH* mRNA levels. Data are expressed as means ± SEM from three biological replicates (one-way ANOVA with post-hoc Tukey test).

First, we found that STAT1 phosphorylation, which is mediated by IFNAR-associated JAK1 and Tyk2 kinases^56^, was totally inhibited in AB8/13 podocytes pretreated for 2 h with 5 μM Ruxolitinib and stimulated for 15 min with 10 ng/ml IFNβ (Fig. 7a). In contrast to phosphorylation of STING, TBK1, and IRF3 detected at 2 h post exposure to nsDNA, treatment with IFNβ alone for 2 h did not detectably stimulate the phosphorylation of STING and IRF3 (Fig. 7a,b). Nevertheless, IFNβ robustly induced expression of IFI16 (Fig. 7c,e) and APOL1 (Fig. 7c,d) at 18 h post treatment. Pretreatment with Ruxolitinib blocked IFI16 protein expression by 93% compared to cells treated only with IFNβ. Expression of IFI16 protein and *IFI16* mRNA was also strongly reduced (by 85% and 88%, respectively) in cells pretreated with Ruxolitinib and transfected with nsDNA (18 h), as compared to cells challenged with nsDNA only (Fig. 7c,g). In contrast, while both nsDNA and exogenous IFNβ stimulated expression of APOL1 protein (Fig. 7c) and *APOL1* mRNA (Fig. 7d,f), Ruxolitinib totally inhibited expression of APOL1 mediated by exogenous IFNβ (Fig. 7c,d) but only moderately reduced expression of APOL1 protein (by 33%) and *APOL1* mRNA (by 49%) triggered by nsDNA (Fig. 7c,f). Together, these results suggest that expression of both APOL1 and IFI16 is mediated by IFNβ produced in response to activation of the STING-TBK1-IRF3 pathway by nsDNA. Subsequent engagement of IFNAR by the released IFNβ activates STAT1, which upregulates transcription of *APOL1* and *IFI16*. However, inhibition of IFNAR signaling by Ruxolitinib reduces but does not abolish APOL1 expression triggered by nsDNA, indicating that APOL1 expression is induced by both IFNβ-dependent (Ruxolitinib sensitive) and independent (Ruxolitinib insensitive) mechanisms, the latter mediated directly by activated IRF3.

### STING knockdown inhibits expression of APOL1 and IFI16 induced by nsDNA but not by exogenous IFNβ

**Figure 8:**
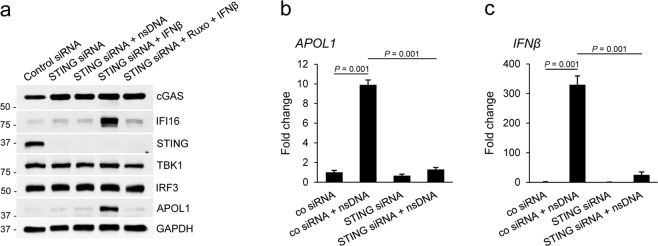
STING knockdown inhibits nsDNA-induced expression of APOL1 and IFI16 but does not affect expression induced by exogenous IFNβ. (**a**) AB8/13 podocytes were transfected for 48 h with a non-targeting control siRNA or siRNA pool targeting STING. Subsequently, sets of the transfected cells were transfected for 18 h with 1 μg ml^−1^ nsDNA, treated for 18 h with IFNβ (10 ng ml^−1^), or pretreated with 5 μM Ruxo for 2 h followed by IFNβ stimulation for 18 h. Expression of indicated proteins was analyzed by immunoblotting. The blot images were obtained from different gels. The blot probed with IFI16 was re-probed with IRF3. Other blot images were cropped from individually probed blots. Full images of the blots are shown in Supplementary Fig. S8. (**b**,**c**) STING knockdown inhibited expression of *APOL1* (**b**) and *IFNβ* mRNA (**c**) in response to nsDNA. AB8/13 podocytes were transfected for 48 h with a non-targeting control siRNA or siRNA pool targeting STING, followed by transfection with 1 μg ml^−1^ nsDNA for 18 h. Expression of *APOL1* and *IFNβ* mRNA was normalized to *GAPDH* mRNA levels. Data are expressed as means ± SEM from three biological replicates (one-way ANOVA with post-hoc Tukey test).

**Figure 9:**
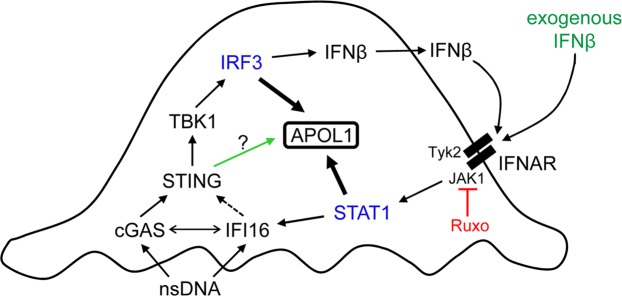
Our proposed model of nsDNA-induced APOL1 expression through engagement of the cGAS/IFI16-STING pathway in human immortalized AB8/13 podocytes. Binding of cytosolic nsDNA by cGAS and IFI16 activates STING, which subsequently activates TBK1. Activated TBK1 phosphorylates IRF3, which promotes transcription of *APOL1* and *IFNβ*. IFNβ released from the cells (or exogenous IFNβ) binds to IFNAR. IFNAR-associated JAK1 and Tyk2 kinases then phosphorylate STAT1, which promotes transcription of *APOL1* and *IFI16*. A putative IFI16-mediated activation of STING is indicated by a dashed arrow. A potential cooperation between cGAS and IFI16 is indicated by a double-headed arrow. Deficient STING phosphorylation observed in cGAS- or IFI16-knockdown cells (Fig. 6) suggests that nsDNA-induced APOL1 expression may be mediated by a phospho-STING-independent pathway, marked by a green arrow. A dual JAK1/JAK2 inhibitor Ruxolitinib (Ruxo) suppresses STAT1 activation and thereby inhibits IFI16 expression and STAT1-mediated APOL1 expression. Since IFNAR-mediated signaling involves JAK1 but not JAK2, our model only depicts the inhibition of JAK1 by Ruxo. Ruxo does not affect IRF3-mediated APOL1 expression.

Multiple innate immune signaling pathways triggered by bacterial cyclic dinucleotides or viral nucleic acids converge on STING^42,44^. Accordingly, we show that nsDNA-triggered APOL1 expression depends on STING and is effectively inhibited by siRNA-mediated STING knockdown in AB8/13 podocytes (Fig. 3a–c). To further evaluate the role of STING in APOL1 expression induced by exogenous IFNβ, we first transfected AB8/13 podocytes with a pool of STING-specific siRNAs or non-targeting control siRNA. After 48 h, we either transfected the cells with nsDNA or treated them with IFNβ in the absence or presence of Ruxolitinib. We observed a potent STING knockdown in cells transfected with STING siRNAs but not in those with the control siRNA (Fig. 8a). We also confirmed that while STING is indispensable for nsDNA-induced expression of APOL1 (Fig. 8a,b) and IFI16 (Fig. 8a), it is not required for expression of these proteins induced by exogenous IFNβ. These data, together with those presented in Fig. 7, confirm that nsDNA stimulates APOL1 expression through a two-step mechanism that involves STING activation. In the first step, nsDNA-triggered activation of the STING-TBK1 axis stimulates IRF3 phosphorylation, which subsequently promotes transcription of *APOL1* and *IFNβ*. In a second step, released IFNβ stimulates transcription of *APOL1* and *IFI16* through IFNAR-mediated signaling that involves activation of JAK1 and STAT1 (Fig. 9). In summary, we conclude that attenuation of APOL1 expression triggered by nsDNA and exogenous IFNβ (derived from sources other than nsDNA-stimulated podocytes) may require suppression of both IFNAR and STING signaling.

## Discussion

APOL1 alleles G1 and G2, found in ~12% of African Americans, are risk factors for developing a spectrum of nondiabetic kidney diseases^3–5^, including LN and LN-ESRD^6,7^. Damage of kidney glomerular podocytes observed in LN^8–10^ suggests that increased expression of APOL1 risk variants in podocytes of SLE patients may contribute to faster progression to LN and LN-ESRD. Because of an incomplete penetrance of the APOL1 risk alleles, environmental factors have been proposed as the key triggers of APOL1 expression and kidney damage in African Americans^18^. Thus, identification of factors that upregulate APOL1 expression in lupus is critical for designing new therapies aimed to prevent or reduce the severity of kidney damage in individuals expressing the APOL1 risk alleles.

Recent study has shown that *APOL1* high-risk genotype does not alter the steady-state levels of *APOL1* mRNA in human glomeruli, as compared to low-risk genotype. However, expression of *APOL1* high-risk genotype, but not low-risk genotype, was associated with kidney injury in NEPTUNE cohort^57^. Since *APOL1* promoter drives the expression of high and low-risk allele RNA, it is conceivable that factors that induce APOL1 expression will have different effects depending on the *APOL1* genotype. Indeed, recent study has shown that APOL1 risk allele mRNA activates protein kinase R^58^, which together with increased expression of APOL1 protein risk variants may contribute to renal toxicity.

Increased levels of blood-circulating nsDNA are common in SLE and LN patients^19,20^. This observation prompted us to propose that nsDNA is a risk factor that contributes to increased APOL1 expression in kidney podocytes.

Here, we demonstrate that exposing human immortalized AB8/13 podocytes (*APOL1* G0/G0) to nsDNA, which represents a mix of genomic DNA sequences of approximately 146 bp, strongly stimulates expression of APOL1 and IFNβ, a type I IFN expressed at high levels in lupus^56,59^. We also demonstrate expression of APOL1 variant G1 in human immortalized MMC111.3 podocytes (APOL1 G1/G1) that we previously established from the urine sample of an African American patient. Together, these observations suggest that expression of both wild-type (G0) and G1 variant of APOL1 as well as IFNβ in response to nsDNA may be mediated by shared cytosolic DNA sensing pathway(s).

Recent reports have demonstrated that cytosolic dsDNA triggers signaling pathways that activate STING^23,60^, which assembles and activates the TBK1-IRF3 signaling cascade^59^ and stimulates expression of type I IFN^61^. However, the sensing of atypically modified RNA by cytosolic RNA-sensing RIG-I-like receptors has been shown to activate STING^47^ and thereby could also contribute to APOL1 expression. We disputed this possibility by demonstrating that nsDNA pretreated extensively with a mix of RNases with different specificities induces a similar pattern of STING pathway activation and APOL1 expression as the RNA-free synthetic dsDNA90^48^ (Supplementary Fig. S9).

Subsequently, we show that siRNA-mediated knockdown of individual components of the STING pathway inhibits APOL1 expression, demonstrating that DNA-sensing receptors that activate the STING pathway likely contribute to APOL1 expression in human AB8/13 podocytes.

Among several DNA-sensing receptors that activate STING^42,44^, cGAS^23^ and IFI16^24^ have been implicated in SLE^25–29^. In this regard, we show that 2′3′-cGAMP, normally synthesized by cGAS in response to dsDNA binding, activates STING and induces APOL1 expression in AB8/13 podocytes, albeit not as robustly as APOL1 expression triggered by nsDNA. Our observation that siRNA-mediated knockdown of cGAS only partially reduces APOL1 expression suggests that additional cytosolic dsDNA sensor(s) in AB8/13 podocytes likely contribute to the upregulation of APOL1 in response to nsDNA. We thus tested if IFI16, a cytosolic/nuclear DNA sensor that activates STING^24^, is expressed in AB8/13 podocytes and can induce APOL1 expression in response to nsDNA. In this regard, we detected relatively low basal levels of IFI16 in unstimulated AB8/13 podocytes that increased significantly in response to nsDNA. cGAS knockdown strongly decreased this effect. Interestingly, cGAS knockdown reduced the basal level of IFI16 by 35% and reduced nsDNA-triggered IFI16 expression by 87%, as compared to cells expressing cGAS and challenged with nsDNA. This suggests that IFI16 expression in AB8/13 podocytes is regulated by the cGAS signaling pathway. Taken together, our observations suggest that cGAS-mediated IFI16 upregulation could increase the sensitivity of the cells to nsDNA and promote higher APOL1 expression.

Thus, expression of APOL1 could be also mediated by IFI16 at its basal expression level in unstimulated AB8/13 podocytes. In this regard, a simultaneous knockdown of cGAS and IFI16 decreased APOL1 expression by 91% in response to nsDNA as compared to control AB8/13 podocytes challenged with nsDNA. This indicates that cGAS and IFI16 are the major nsDNA sensors in AB8/13 podocytes and are both required for maximal APOL1 expression. We confirmed this observation in cGAS^−/−^ knockout AB8/13 podocytes by demonstrating that IFI16 expressed at low basal levels was sufficient to induce suboptimal APOL1 expression in response to nsDNA. Although the mechanism by which relatively low levels of IFI16 upregulate APOL1 expression in human AB8/13 podocytes in response to nsDNA is unknown, the results from this study suggest that this pathway is STING-mediated and phosphorylation-independent. It is conceivable that even at low levels, IFI16 may elicit significant responses to nsDNA of ~146 bp long, which corresponds to dsDNA of ~150 bp previously reported as optimal for engaging IFI16^35^.

Consistent with previous observation^62^, we found that nsDNA induces STING phosphorylation and its subsequent degradation in AB8/13 podocytes. In contrast, knockdown of IFI16, and to a lesser extent of cGAS, reduces accumulation of total STING protein without significantly affecting its phosphorylation. Whether the reduced expression of STING in IFI16-knockdown cells in response to nsDNA reflects STING activation and APOL1 upregulation through an alternative phosphorylation-independent pathway^63^ remains to be determined. One possible mechanism could involve posttranslational modifications of STING. In this regard, K63-linked ubiquitination has been shown to positively regulate STING activity^64^.

Interestingly, in cGAS-knockout cells, nsDNA does not affect STING expression despite causing a moderate increase in APOL1 expression. This suggests that in the absence of cGAS, IFI16 can still stimulate APOL1 expression. Indeed, IFI16 knockdown in these cells almost completely eliminates APOL1 expression. The constant expression of STING in cGAS-knockout and IFI16 knockdown cells challenged with nsDNA may be due to the depletion of nsDNA sensors in these cells. Thus, constant levels of STING in unstimulated and nsDNA-induced cGAS- knockout cells that express APOL1 supports the involvement of an IFI16-mediated pathway in APOL1 expression. Although the nature of this pathway is currently unknown, we speculate that it may involve a signaling complex consisting of nsDNA, IFI16, and STING assembled on the ER.

While we did not investigate the precise mechanism underlying the interaction between cGAS and IFI16 that appears to converge at STING activation, our results are consistent with recent findings showing that cooperation between IFI16 and cGAS is required for optimal DNA sensing in human macrophages^37^ and in human keratinocytes^36^. Together, these results and our observations suggest that the cross-talk between IFI16 and cGAS may represent a common mechanism adopted by different cell types to regulate innate responses to cytosolic DNA.

Stimulation of APOL1 and IFNβ expression in response to cytosolic nsDNA suggests that APOL1 expression could be mediated, at least in part, by IFNβ. To assess the contribution of IFNβ to APOL1 expression, we used Ruxolitinib, a pharmacological inhibitor of JAK1/JAK2 kinases that blocks IFN signaling^53^ by inhibiting IFNAR-associated JAK1 kinase and STAT1^56^. Since STAT1 promotes transcription of both *APOL1*^17^ and *IFI16*^65^, total inhibition of nsDNA-triggered IFI16 expression by Ruxolitinib implicates IFNβ as a direct inducer of IFI16 expression in AB8/13 podocytes.

Interestingly, a moderate inhibition of APOL1 expression by Ruxolitinib is consistent with the activation of an additional IFNβ-independent pathway that promotes APOL1 expression. In this regard, we demonstrate that nsDNA triggers the IFNβ-independent cGAS/IFI16-STING signaling cascade and stimulates IRF3-dependent expression of APOL1. However, IRF3-mediated IFNβ expression^66^ may further boost APOL1 expression and increase IFI16 levels through IFNβ-dependent STAT1 activation. It is likely that higher levels of IFI16 in AB8/13 podocytes may increase the cross-talk with cGAS and further stimulate expression of APOL1 and IFNβ through a positive feedback mechanism.

In summary, we demonstrate that in human AB8/13 podocytes, nsDNA activates the cGAS/IFI16-STING signaling cascade and triggers APOL1 expression through IFNβ-independent and dependent pathways. We also show that STING knockdown abolishes the expression of APOL1 and IFNβ in AB8/13 podocytes in response to nsDNA. However, in the presence of exogenous IFNβ, induction of APOL1 expression still occurred in STING-knockdown cells via IFNAR-mediated signaling and STAT1 activation. This suggests that the blocking of both IFNβ-dependent (for instance, with JAK inhibitors) and independent (with STING inhibitors) pathways may be the optimal therapeutic intervention to prevent excessive APOL1 expression in podocytes and possibly mitigate progression to LN in SLE patients carrying both APOL1 risk alleles.

## Methods

### Cell culture

Human conditionally immortalized glomerular podocytes AB8/13^41^ and human urine-derived MMC111.3 podocytes were cultured at 33 °C in full RPMI 1640 media supplemented with 10% fetal bovine serum (Clontech), insulin-transferrin-selenium (ITS, Invitrogen), and gentamycin (50 μg/ml). MMC111.3 podocytes homozygous for *APOL1* G1 variant (G1/G1) were established from the urine of an African American donor. The studies were reviewed and approved by the Meharry Medical College Institutional Review Board (protocol FWA00003675). Informed consent was obtained from all participants. All research was performed in accordance with the Meharry Medical College guidelines and regulations. *APOL1* G1 mutations were confirmed by DNA sequencing. The cells were also characterized by immunofluorescence and RT-PCR for the expression of podocyte markers synaptopodin, nephrin, podocalyxin, and WT1.

### Generation of cGAS^−/−^ AB8/13 cells

AB8/13 podocytes were cultured in six-well plates (2.5 × 10^5^ cells/well) and transfected with the cGAS CRISPR double nickase plasmid (1–3 μg/ml, Santa Cruz Biotechnology) using the jetPrime reagent (VWR) following manufacturer’s protocol. After 48 h, the cells were treated with puromycin (5 μg/ml) for three weeks to select for cells that incorporated the plasmid. The culture media were replaced every 2–3 days with fresh full culture media supplemented with puromycin. Selected AB8/13 cGAS^−/−^ podocytes were tested by immunoblotting and RT-PCR to confirm cGAS knockout.

### Preparation of nsDNA from AB8/13 Cells

AB8/13 cells were cultured in T75 flasks until they reached 80–90% confluency. The cells were then trypsinized, resuspended in cold PBS, and pelleted by centrifugation. Pelleted cells were subsequently resuspended in cold PBS and pelleted again. Nuclei were isolated using the EZ Nucleosomal DNA Prep kit (Zymo Research) following manufacturer’s protocol. In short, pelleted cells were resuspended in Nuclei Prep buffer, pelleted and subsequently resuspended in Atlantis Digestion buffer, and treated with Atlantis dsDNase for 30 min at 42 °C. Contaminating RNA was removed by digestion with an RNase cocktail containing RNase A and RNAse T1 (Invitrogen). The nsDNA was further purified according to manufacturer’s instructions. The integrity of the nsDNA preparation was confirmed by resolving the sample in 2% agarose gel followed by staining with ethidium bromide.

### Transfection of nsDNA and cGAMP

AB8/13 podocytes seeded in six-well plates (3.5 × 10^5^ cells/well) were transfected with indicated concentrations of nsDNA or with 4 μg/ml of 2′3′-cGAMP (Invivogen) using the jetPrime transfection reagent (VWR) according to manufacturer’s instructions. Mock transfections were performed using the jetPrime reagent only.

### Preparation and transfection of synthetic dsDNA90

A sense strand of 90mer oligonucleotide (Life Technologies) (5′-TACAGATCTACTAGTGATCTATGACTGATCTGTACATGATCTACATACATACA GATCTACTAGTGATCTATGACTGATCTGTACATGATCTACA-3′)^48^ was annealed to an antisense strand to create the synthetic dsDNA90. AB8/13 podocytes were transfected with 0.5 or 1 μg/ml of synthetic dsDNA90 using the jetPrime reagent.

### siRNA transfection

AB8/13 podocytes were seeded in six-well plates at 2.5 × 10^5^ cells/well for single siRNA transfection or at 3.8 × 10^5^ cells/well for double siRNA transfection. Cells were transfected with pools of siRNAs specifically targeting IFI16, cGAS, STING, TBK1, and IRF3, as well as non-targeting control siRNA (Santa Cruz Biotechnology) using the jetPrime reagent. On day 1, cells were transfected with 10 nM siRNAs and transfection was repeated on day 2. On day 3, the culture media were replenished, and cells were left to recover for 8 h before transfection with 1 μg/ml of nsDNA.

### Ruxolitinib treatment

AB8/13 podocytes seeded in six-well plates (3.5 × 10^5^ cells/well) were pretreated for 2 h with 5 μM Ruxolitinib in DMSO or with DMSO only. The cells were subsequently treated with 10 ng/ml human IFNβ (PeproTech) or transfected with 1 ug/ml nsDNA according to experimental protocols detailed in the legends for Figs 7 and 8.

### Western blot analysis and antibodies

Total cell extracts were harvested in RIPA buffer (Life Technologies) supplemented with a cocktail of protease and phosphatase inhibitors (Life Technologies). Lysates were incubated on ice for 30 min and then clarified by centrifugation. Total protein concentration was measured using the micro BCA protein assay kit (Life Technologies). Protein lysates (25–40 μg protein/lane) were resolved by 10% SDS-PAGE. Resolved proteins were transferred to a nitrocellulose membrane (Bio-Rad). The membrane was blocked with 5% milk in 0.1% TBST (0.1% Tween 20, 20 nM Tris, 150 nM NaCl) and incubated at 4 °C overnight with primary antibodies. The following antibodies were used: APOL1 (Sigma, HPA018885, 1:5000), cGAS (Cell Signaling, D1D3G, 1:1000), IFI16 (Santa Cruz, sc-8023, 1:200), STING (Cell Signaling, D2P2F, 1:1000), P-STING (S366, Cell Signaling, D7C3S, 1:1000), TBK1 (Cell Signaling, D1B4, 1:500), P-TBK1 (S172, Cell Signaling, D52C2, 1:500), IRF3 (Cell Signaling, D6I4C, 1:1000), P-IRF3 (S386, Sigma, ABE501, 1:1000), STAT1 (Santa Cruz, sc-464, 1:500), P-STAT1 (Y701, Santa Cruz, sc-136229, 1:500), and GAPDH (Santa Cruz, sc-25778, 1:15000). Nitrocellulose-immobilized proteins were detected using the WesternBright ECL system (Advansta). Intensities of indicated protein bands were quantified by densitometric scanning using ChemiDoc Imager and Quantity One software (Bio-Rad), and normalized against the intensities of respective GAPDH protein bands.

### Quantitative real-time PCR (qRT-PCR)

RNA was extracted from the cells using the Quick RNA MiniPrep kit (Zymo Research). RNA preparations were then treated with TURBO DNase (Life technologies) and purified according to manufacturer’s protocol. cDNA was synthesized using the iScript cDNA Synthesis Kit (Bio-Rad). Real-time PCR was performed on a CFX-96 instrument (Bio-Rad) using the iQ SYBR Green Super Mix (Bio-Rad). mRNA expression was normalized to *GAPDH* mRNA levels. PCR primers (Life Technologies) used in this study are as follows:

APOL1 (FWD): 5′-GCTTTGCTGAGAGTCTCTGTCC-3′

APOL1 (REV): 5′-GGGCTTACTTTGAGGATCTCCAG-3′

IFNβ (FWD): 5′-CTTGG ATTCCTACAAAGAAGCAGC-3′

IFNβ (REV): 5′-TCCTCCTTCTGGAACTGCTGCA-3′

STING (FWD): 5′-CCTGAGTCTCAGAACAACTGCC-3′

STING (REV): 5′-GGTCTTCAAGCTGCCCACAGTA-3′

TBK1 (FWD): 5′-CAACCTGGAAGCGGCAGAGTTA-3′

TBK1 (REV): 5′-ACCTGGAGATAATCTGCTGTCGA-3′

IRF3 (FWD): 5′-TCTGCCCTCAACCGCAAAGAAG-3′

IRF3 (REV): 5′-TACTGCCTCCACCATTGGTGTC-3′

cGAS (FWD): 5′-AGGAAGCAACTACGACTAAAGCC-3′

cGAS (REV): 5′-CGATGTGAGAGAAGGATAGCCG-3′

IFI16 (FWD): 5′-GATGCCTCCATCAACACCAAGC-3′

IFI16 (REV): 5′-CTGTTGCGTTCAGCACCATCAC-3′

GAPDH (FWD): 5′-GAAGGTGAAGGTCGGAGT-3′

GAPDH (REV): 5′-GAAGATGGTGATGGGATTTC-3′

### Statistical analysis

Data are expressed as means ± SEM. For comparison of means between two groups, the unpaired two-tailed Student’s *t*-test was performed. Statistical comparisons between four groups were performed using one-way analysis of variance (ANOVA) with post-hoc Tukey test. A P-value of <0.05 was considered statistically significant.

## Supplementary information

Nucleosomal dsDNA Stimulates APOL1 Expression in Human Cultured Podocytes by Activating the cGAS/IFI16-STING Signaling Pathway

## Data Availability

The datasets generated and/or analyzed during this study are available from the corresponding author upon reasonable request.
