# Supplementary material for: Nucleosomal dsDNA Stimulates APOL1 Expression in Human Cultured Podocytes by Activating the cGAS/IFI16-STING Signaling Pathway
Source: Sci Rep. 2019 Oct 29;9:15485. doi: 10.1038/s41598-019-51998-w (PMC6820523; doi:10.1038/s41598-019-51998-w)

Supplementary Information

Nucleosomal dsDNA Stimulates APOL1 Expression in Human Cultured Podocytes by Activating the cGAS/IFI16-STING Signaling Pathway

Shamara E. Davis[1], Atanu Khatua[1] & Waldemar Popik[1,2]*

[1]Meharry Medical College, Center for AIDS Health Disparities Research, Department of Microbiology and Immunology, and [2]Department of Internal Medicine, 1005 D. B. Todd Blvd, Nashville, TN 37028, USA.

Supplementary Figure S1

Fig. 1b APOL1

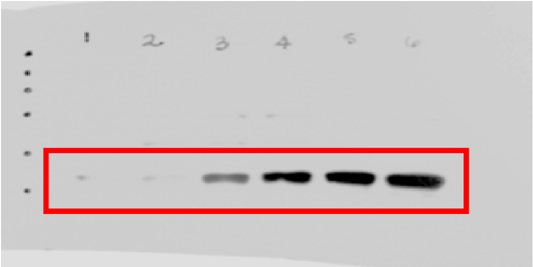

Fig. 1b GAPDH

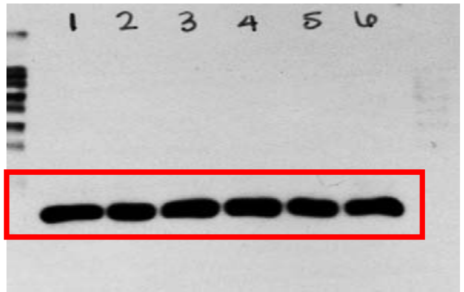

Fig. 1e APOL1

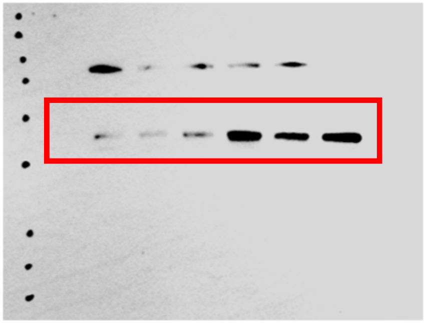

Fig. 1e GAPDH

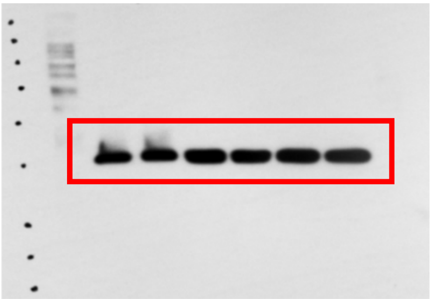

Supplementary Figure S2

Fig. 2a P-STING/STING

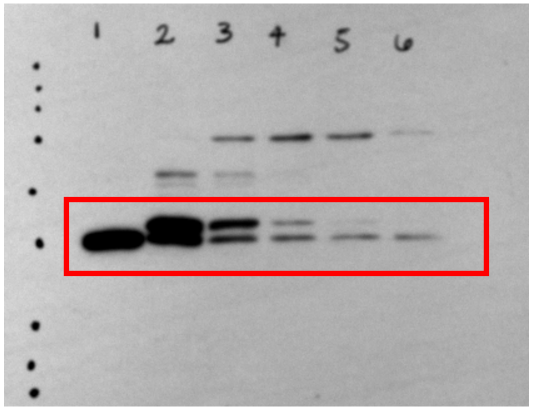

Fig. 2a P-TBK1

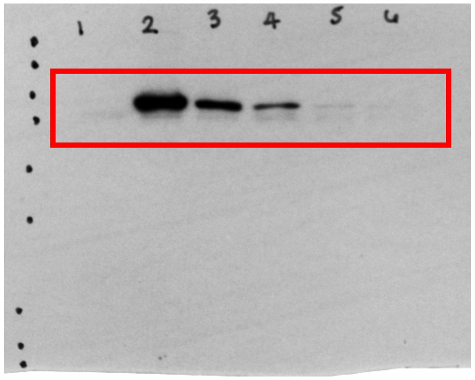

Fig. 2a TBK1

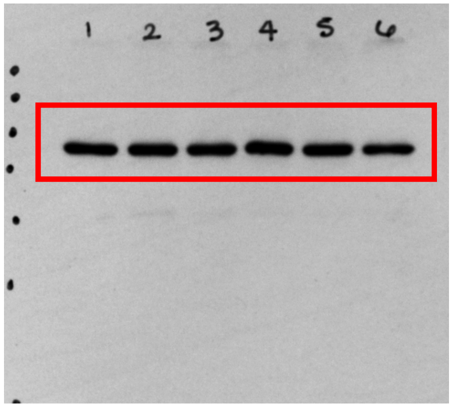

Fig. 2a P-IRF3

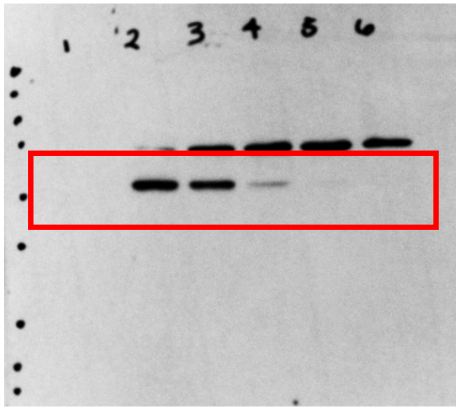

Fig. 2a IRF3

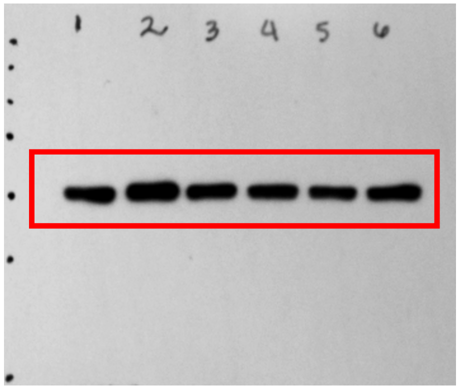

Fig. 2a APOL1

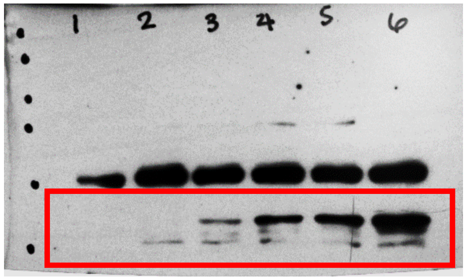

Fig. 2a GAPDH

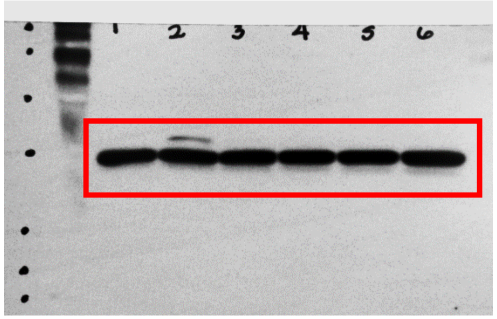

Supplementary Figure S3

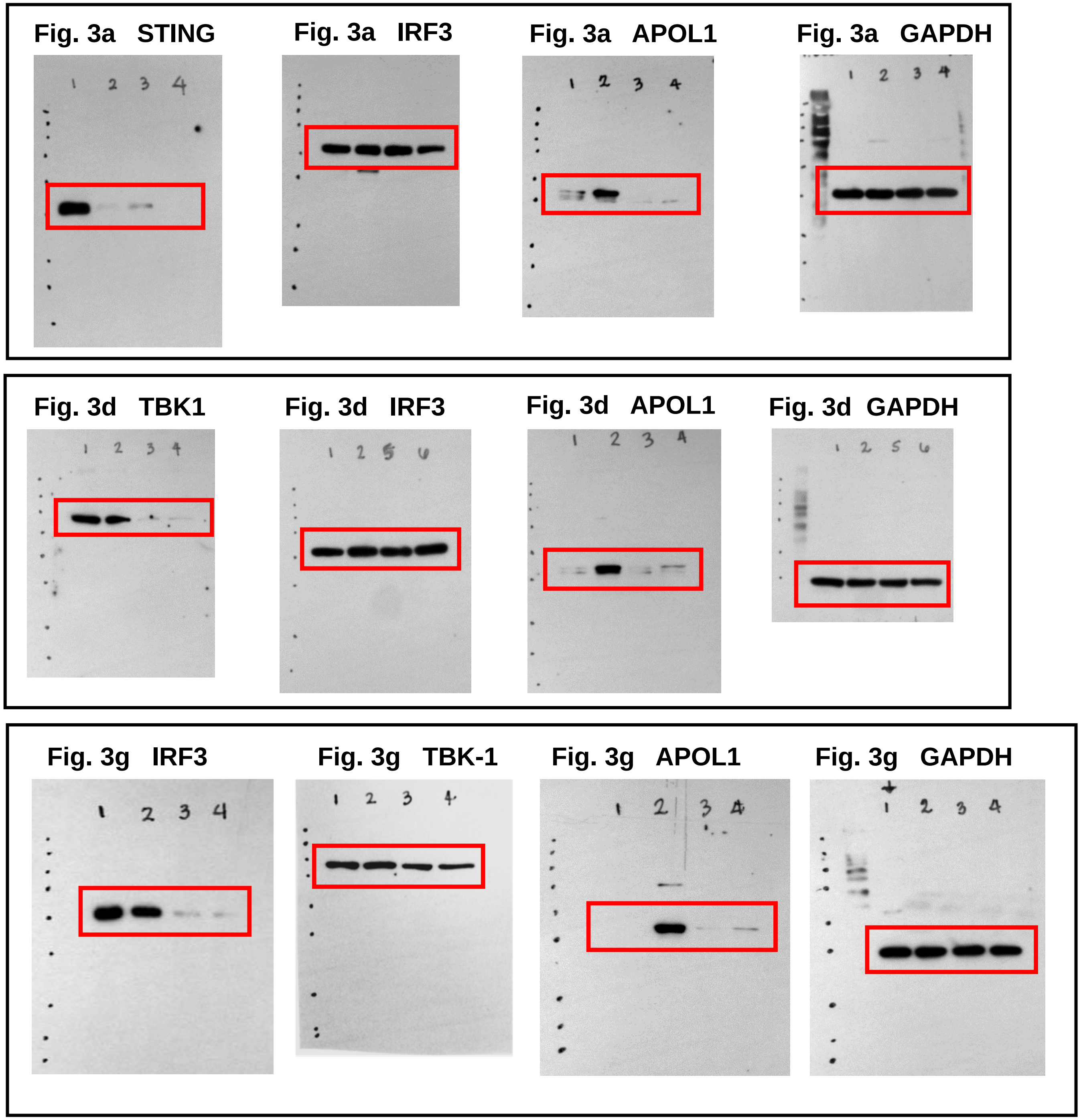

Supplementary Figure S4

Fig. 4 P-STING

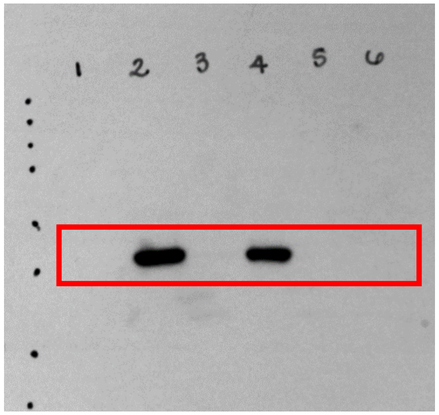

Fig. 4 STING

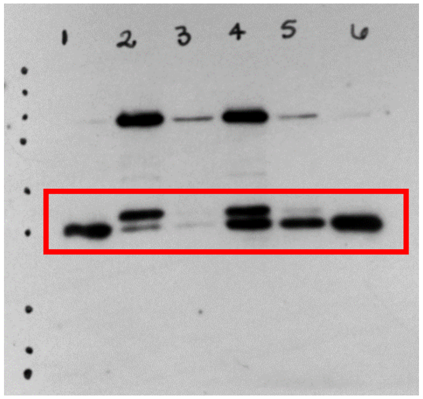

Fig. 4 P-TBK1

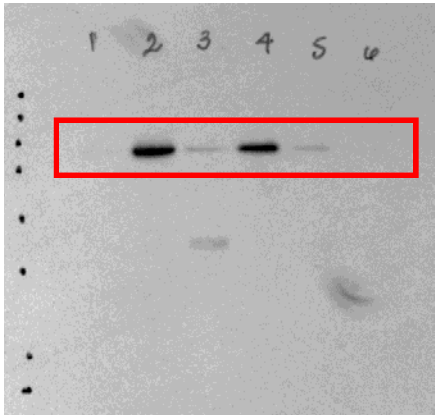

Fig. 4 TBK1

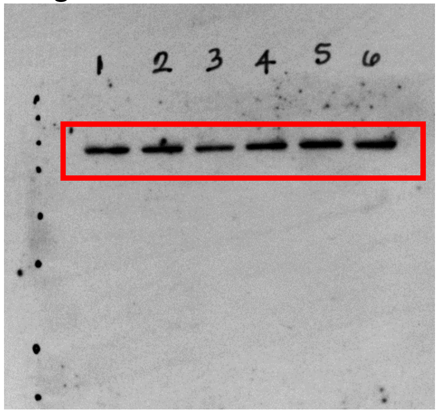

Fig. 4 P-IRF3

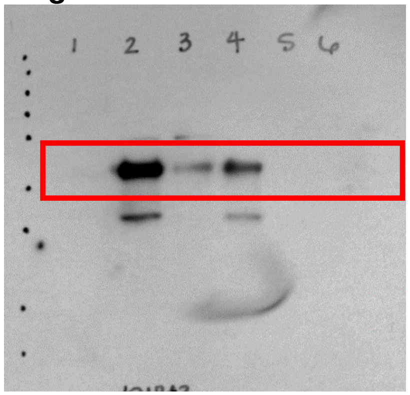

Fig. 4 IRF3

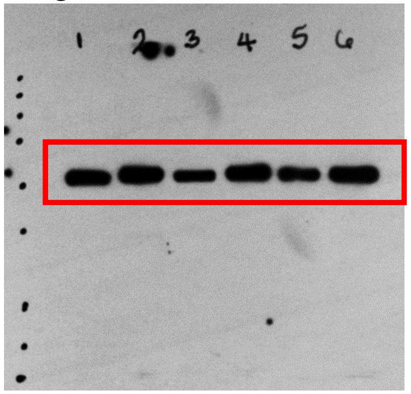

Fig. 4 APOL1

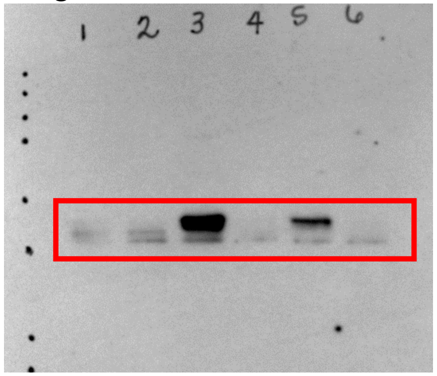

Fig. 4 GAPDH

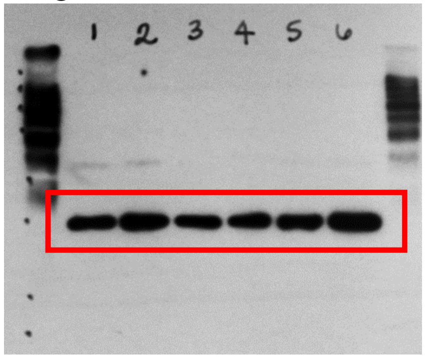

Supplementary Figure S5

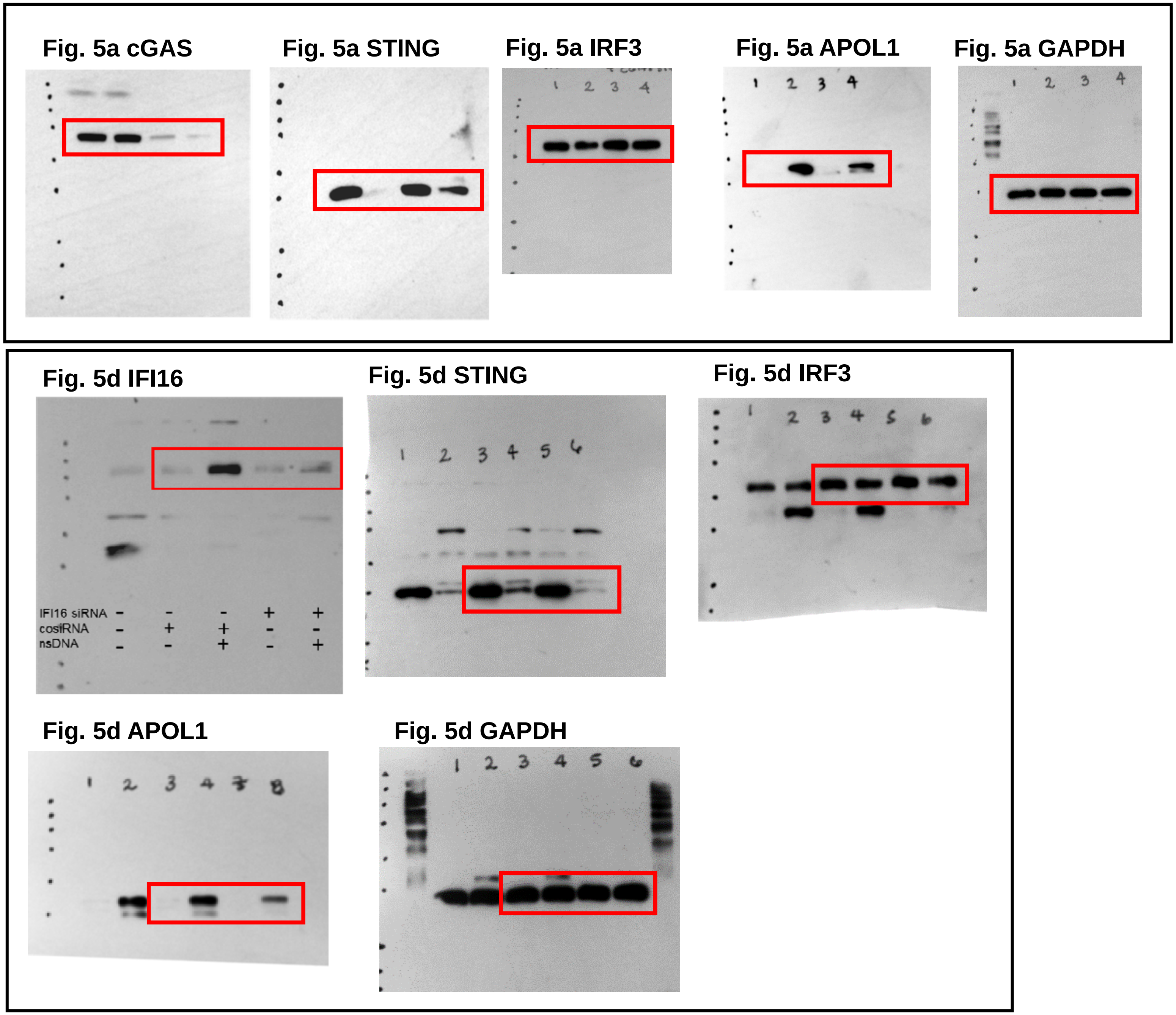

Supplementary Figure S6

Fig. 6a cGAS

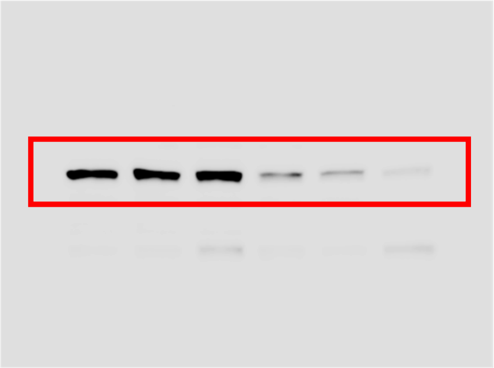

Fig. 6a IFI16

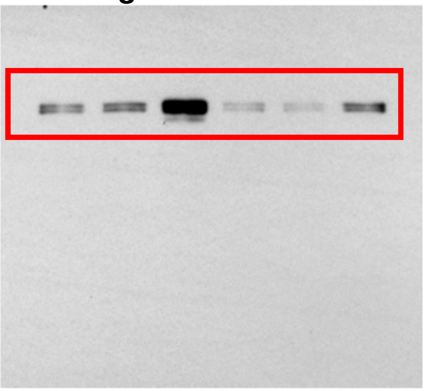

Fig. 6a P-TBK1

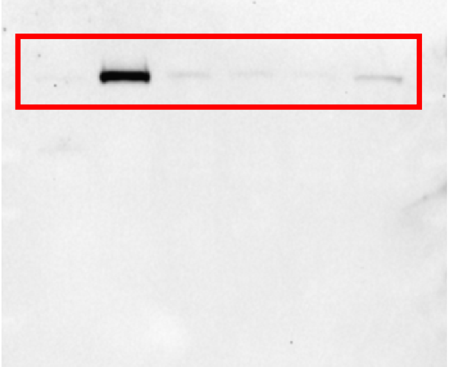

Fig. 6a TBK1

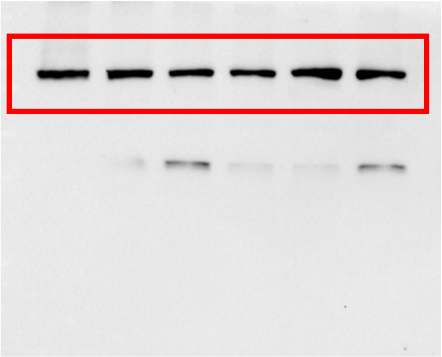

Fig. 6a P-STING

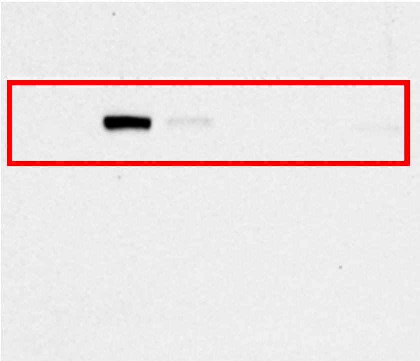

Fig. 6a STING

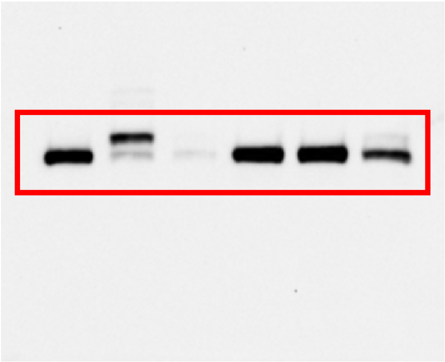

Fig. 6a APOL1

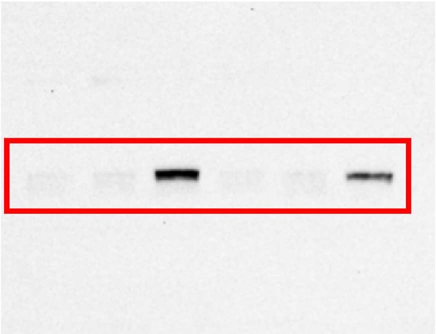

Fig. 6a GAPDH

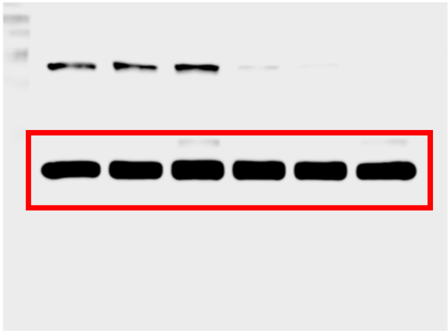

Fig. 6a P-IRF3

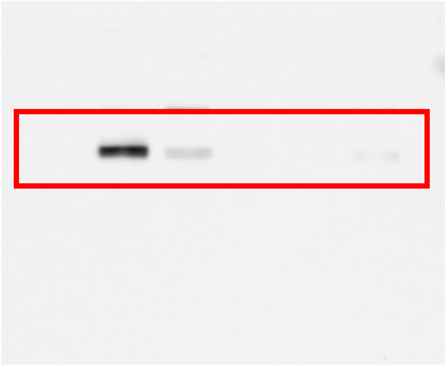

Fig. 6a IRF3

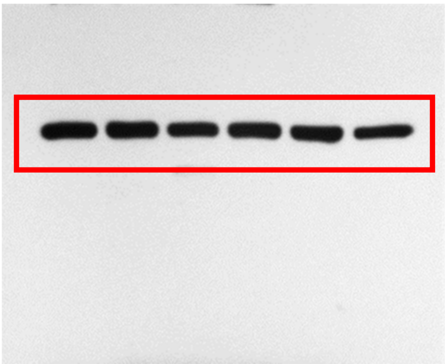

Fig. 6a. Left panel: control siRNA and cGAS siRNA

Supplementary Figure S6 cont.

Fig. 6a cGAS

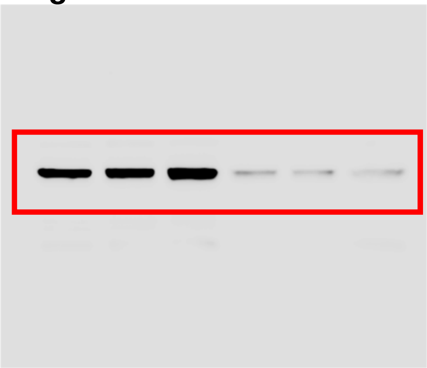

Fig. 6a IFI16

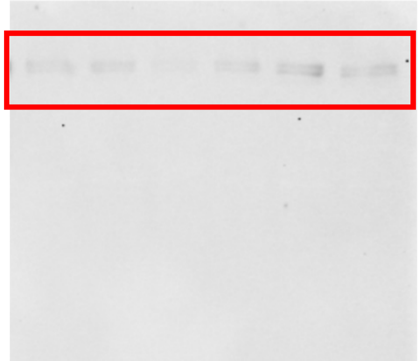

Fig. 6a P-TBK1

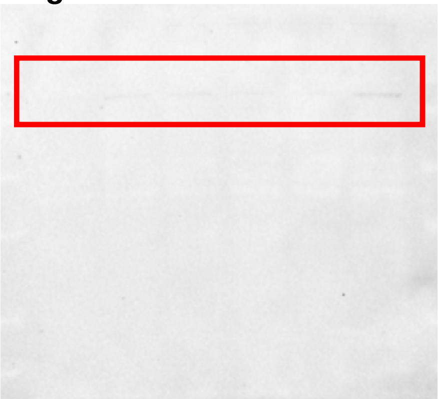

Fig. 6a TBK1

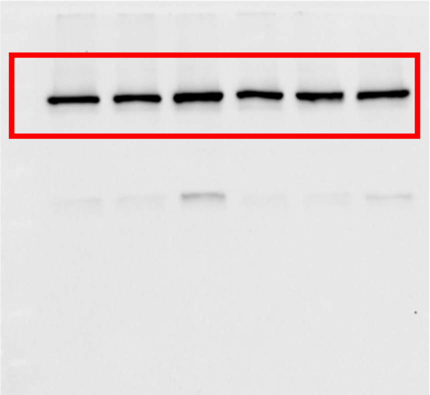

Fig. 6a P-STING

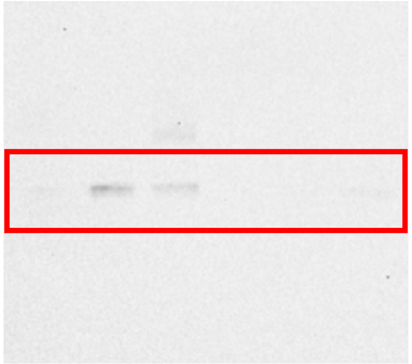

Fig. 6a STING

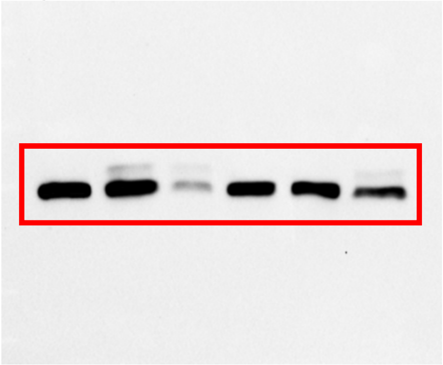

Fig. 6a APOL1

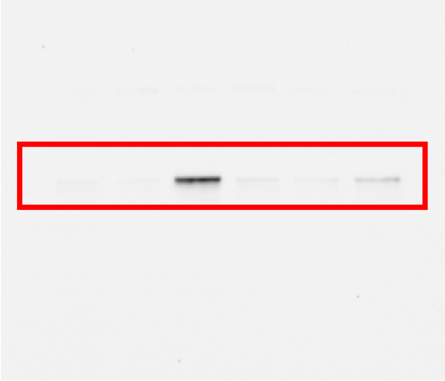

Fig. 6a GAPDH

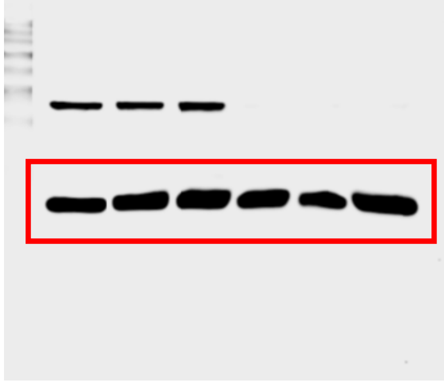

Fig. 6a P-IRF3

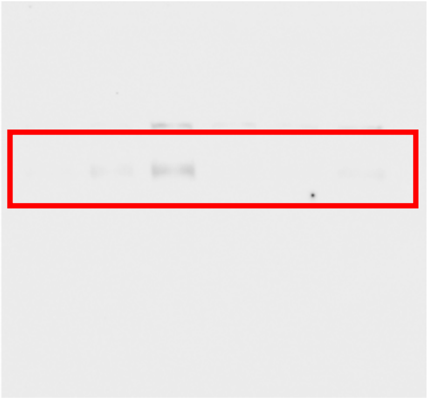

Fig. 6a IRF3

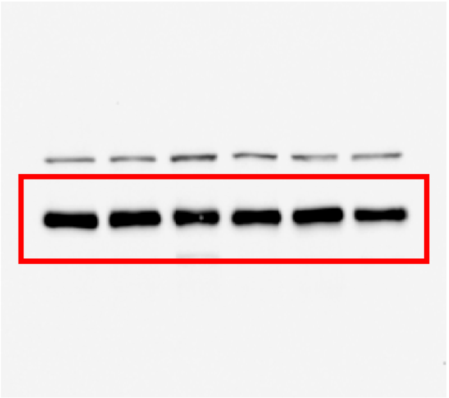

Fig. 6a. Right panel: IFI16 siRNA and cGAS siRNA + IFI16 siRNA

Supplementary Figure S6 cont.

Fig. 6b cGAS

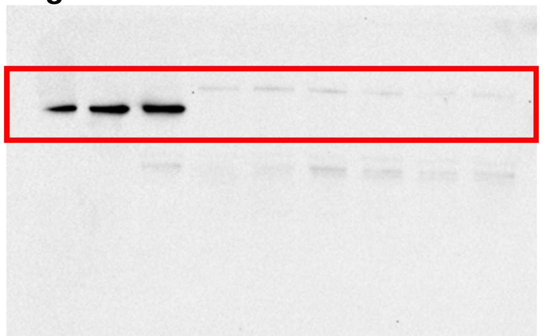

Fig. 6b IFI16

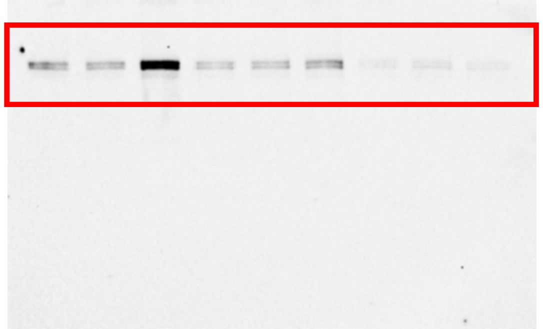

Fig. 6b APOL1

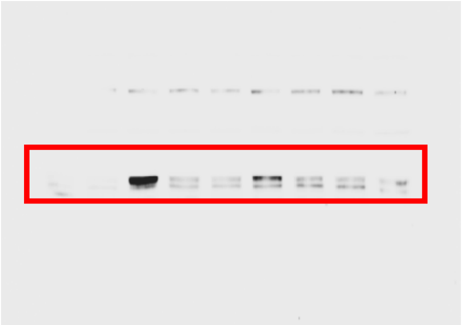

Fig. 6b STING

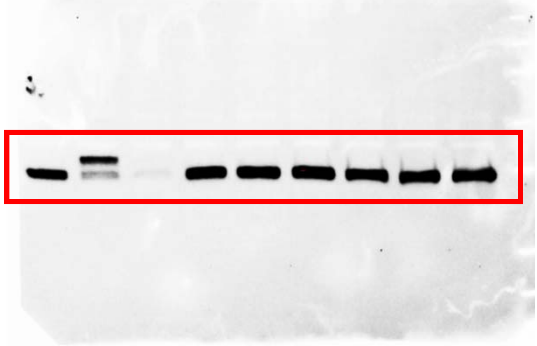

Fig. 6b P-STING

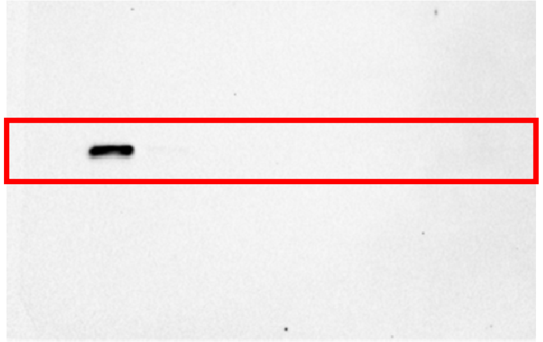

Fig. 6b GAPDH

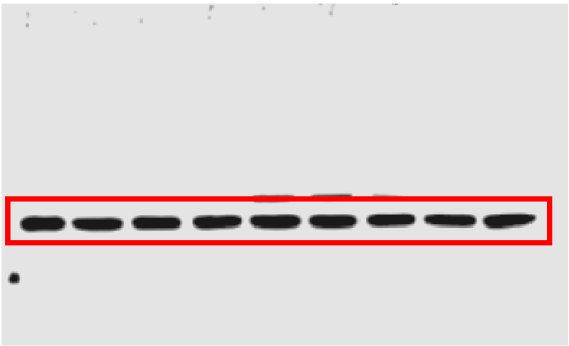

Fig. 6b P-IRF3

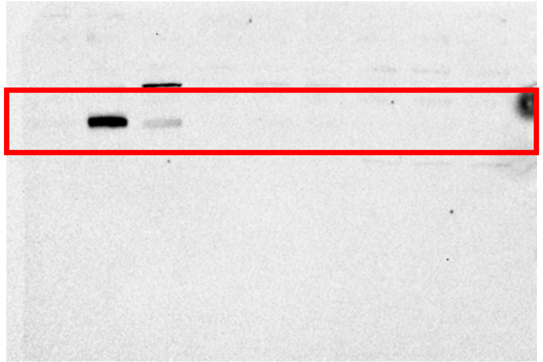

Fig. 6b IRF3

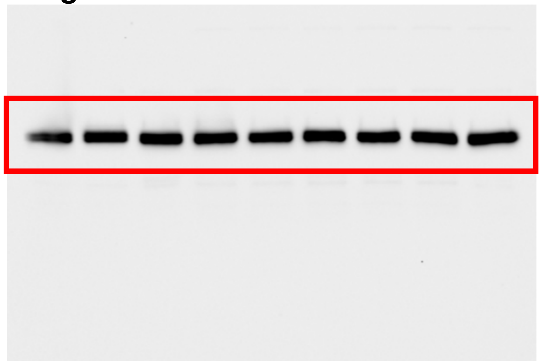

Supplementary Figure S7

Fig. 7a P-STAT1

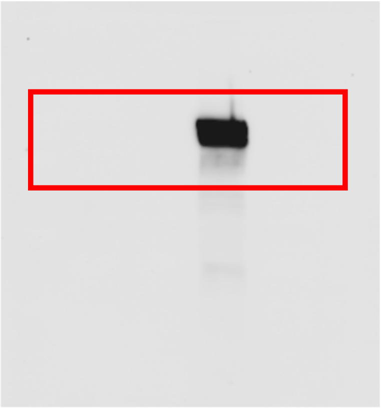

Fig. 7a STAT1

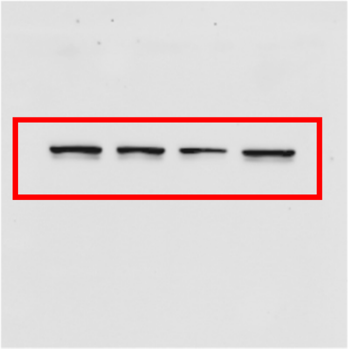

Fig. 7a P-IRF3

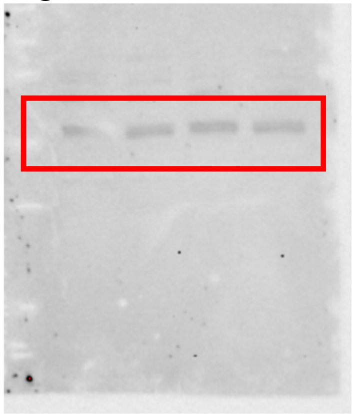

Fig. 7a IRF3

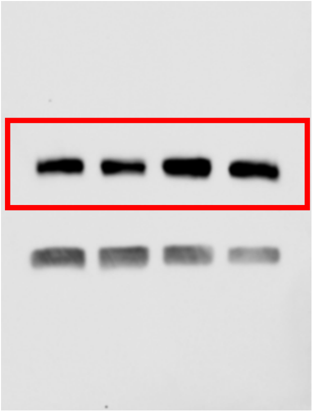

Fig. 7a P-STING

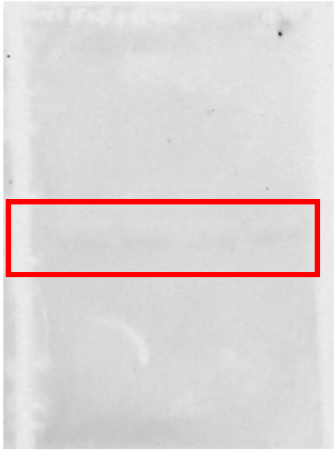

Fig. 7a STING

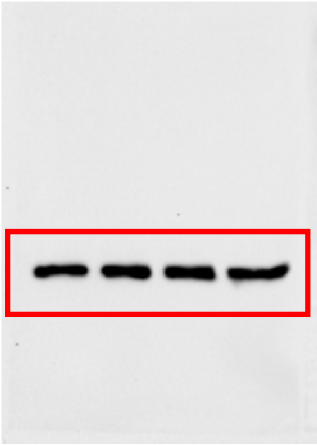

Fig. 7a GAPDH

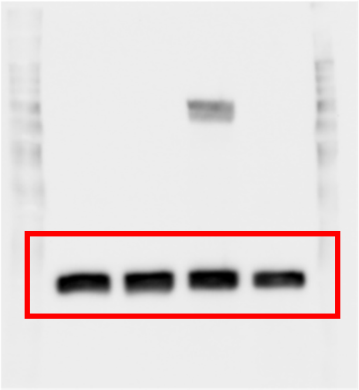

Supplementary Figure S7 cont.

Fig. 7b cGAS

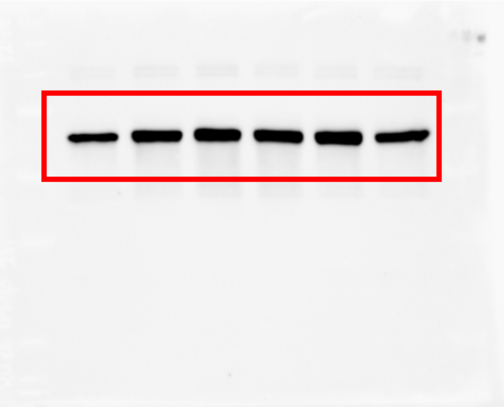

Fig. 7b IFI16

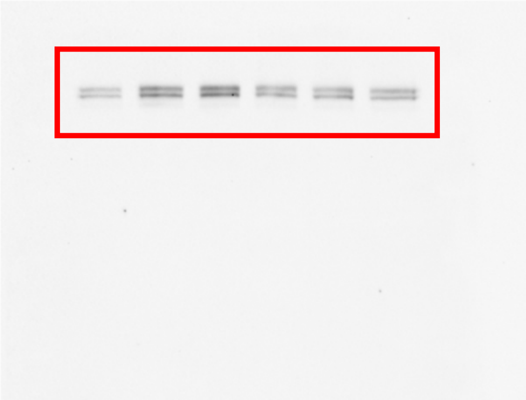

Fig. 7b P-STING

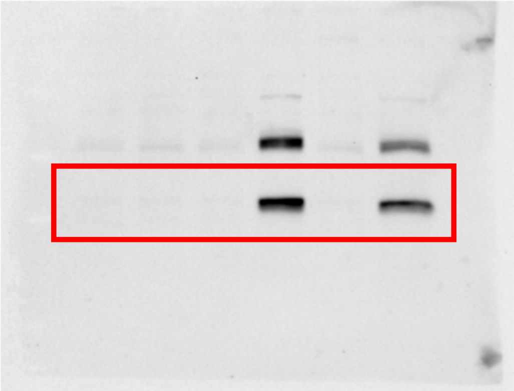

Fig. 7b STING

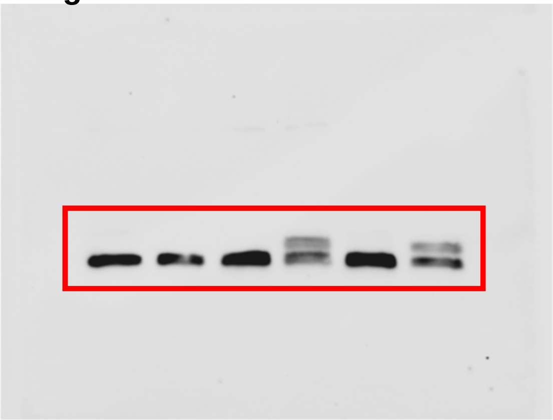

Fig. 7b P-TBK1

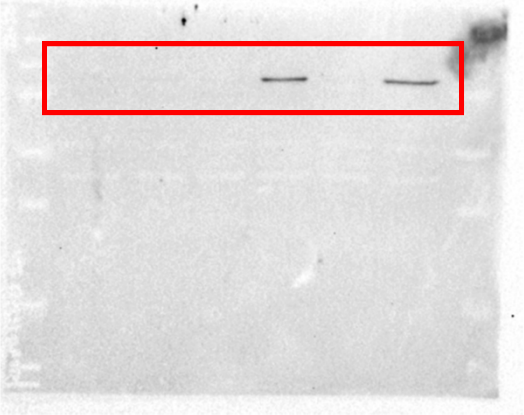

Fig. 7b TBK1

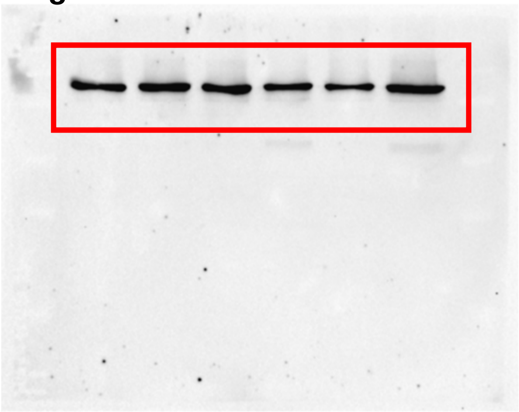

Fig. 7b P-IRF3

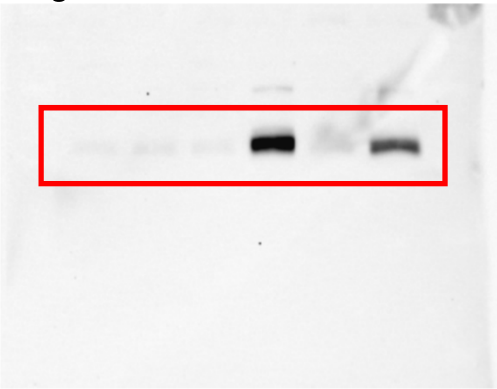

Fig. 7b IRF3

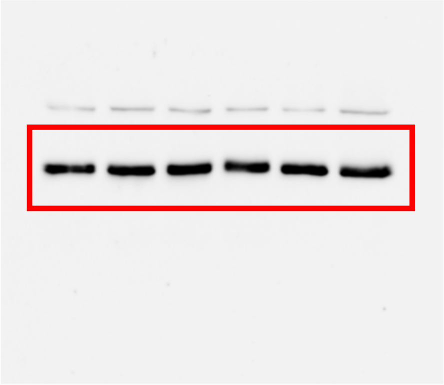

Fig. 7b APOL1

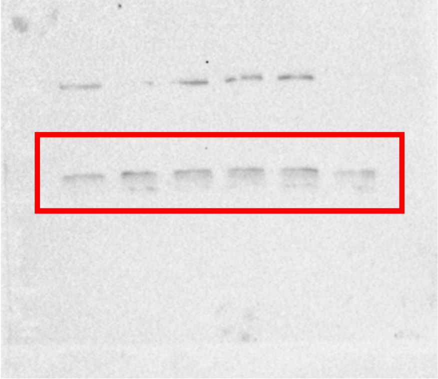

Fig. 7b GAPDH

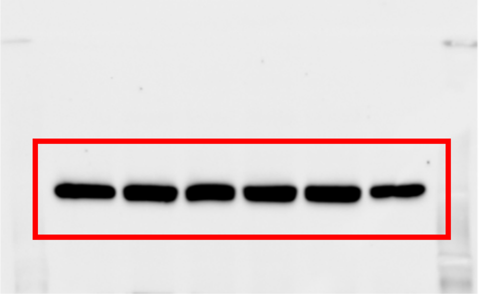

Supplementary Figure S7 cont.

Fig. 7c cGAS

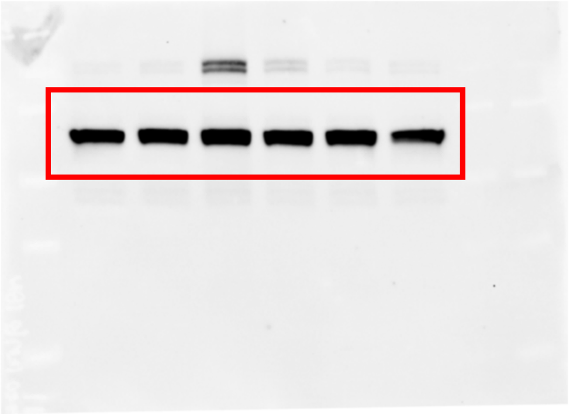

Fig. 7c IFI16

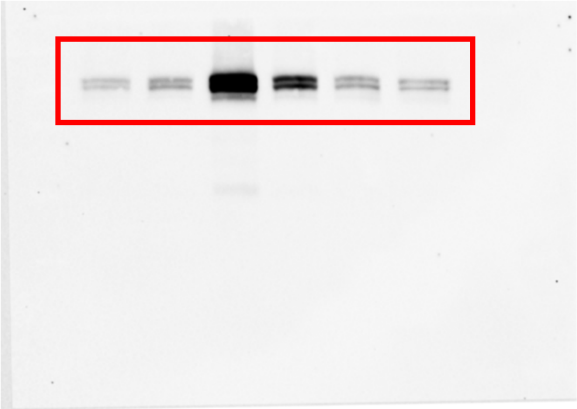

Fig. 7c STING

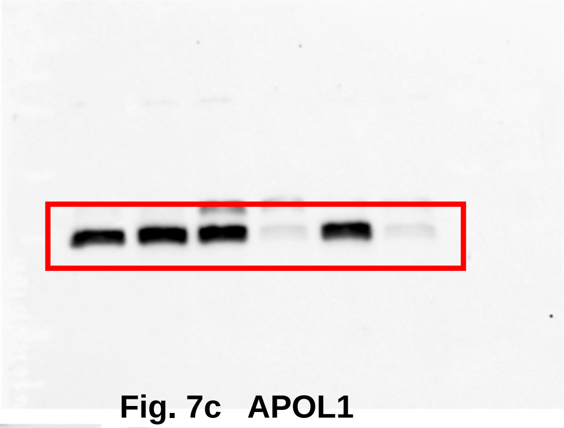

Fig. 7c TBK1

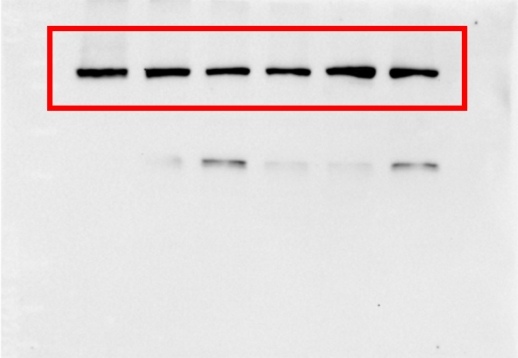

Fig. 7c IRF3

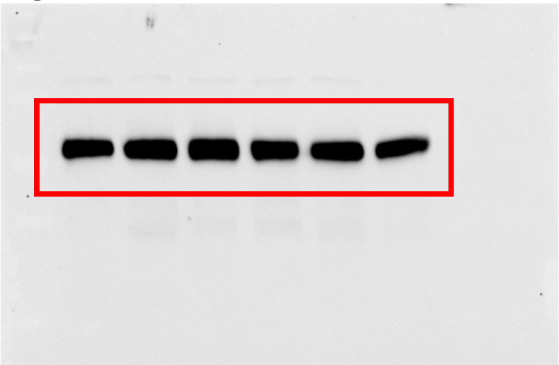

Fig. 7c APOL1

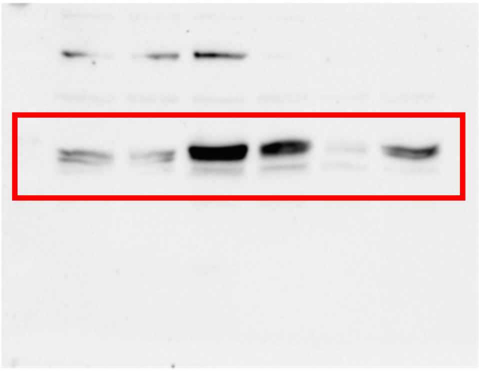

Fig. 7c GAPDH

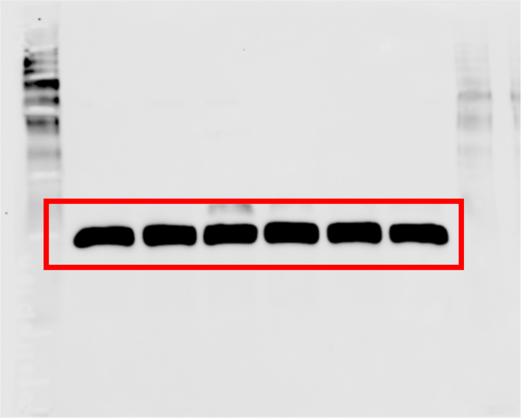

Supplementary Figure S8

Fig. 8a cGAS

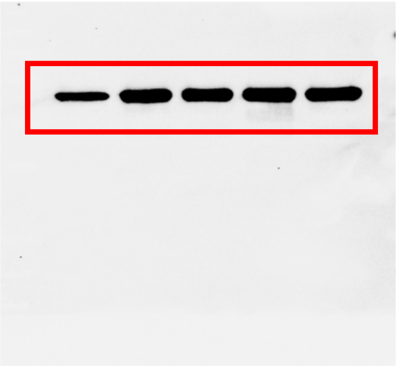

Fig. 8a IFI16

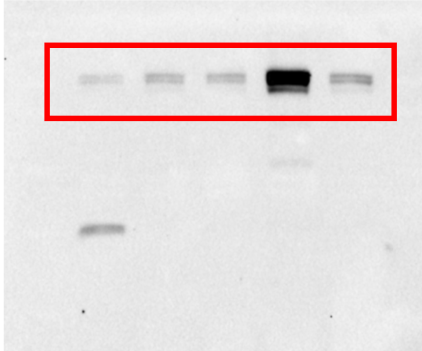

Fig. 8a STING

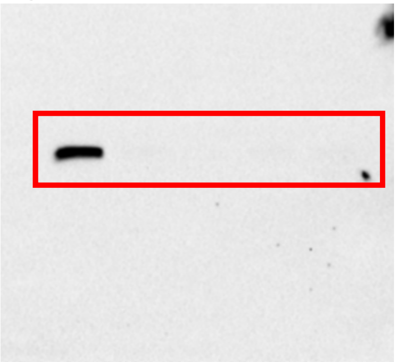

Fig. 8a TBK1

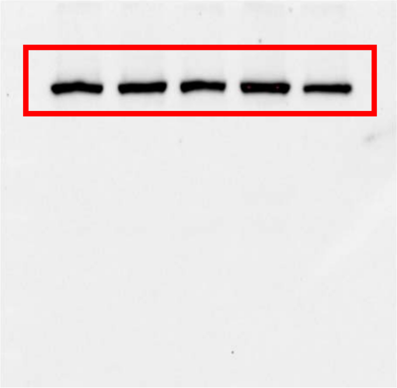

Fig. 8a IRF3

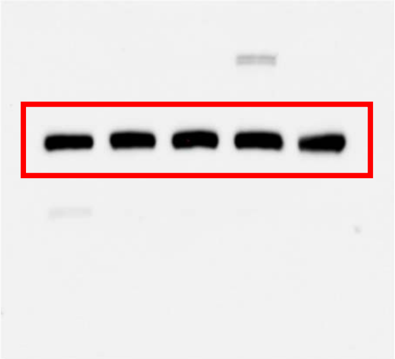

Fig. 8a APOL1

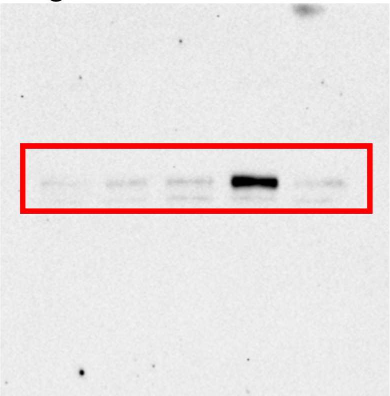

Fig. 8a GAPDH

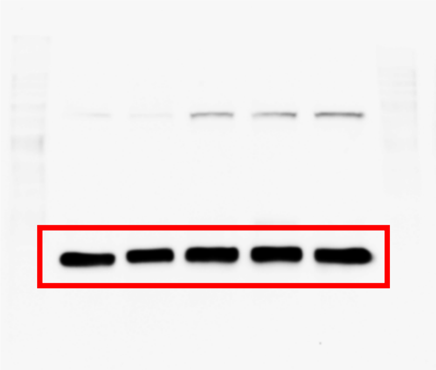

Supplementary Figure S9

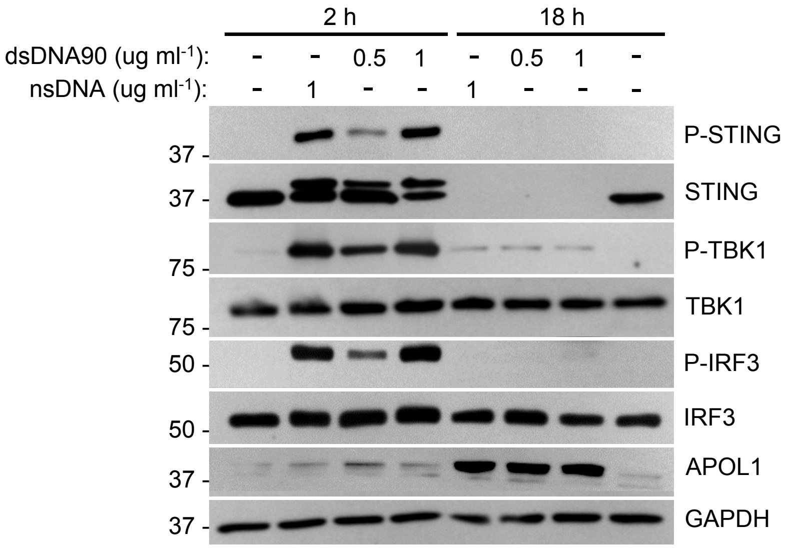

Supplementary Figure S9. Cellular RNA was not detected in the nsDNA samples used in this study. AB8/13 podocytes were mock transfected (-) or transfected with 1 μg ml^{-1} nsDNA that had been treated with a mix of RNase A and RNase T1 to remove potential RNA contamination. In a parallel experiment, the cells were transfected with two doses of RNA-free synthetic dsDNA90 (0.5 or 1 μg ml^{-1}) for 2 h or 18 h. Expression of indicated proteins was analyzed by immunoblotting. Protein size markers (kDa) are indicated. The blot probed for P-STING was re-probed for IRF3. The other blot images were cropped from individually probed blots. Full images of the blots are shown in Supplementary Figure S9 cont. (shown below).

Supplementary Figure S9 cont. Full images

Fig. S9 P-STING

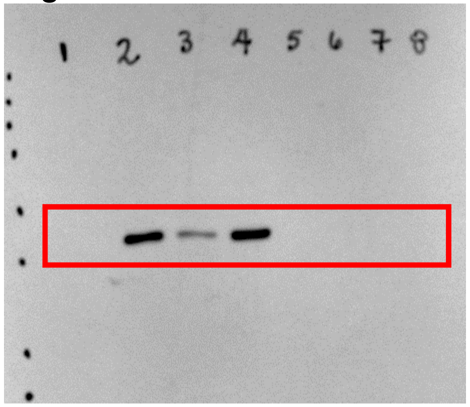

Fig. S9 STING

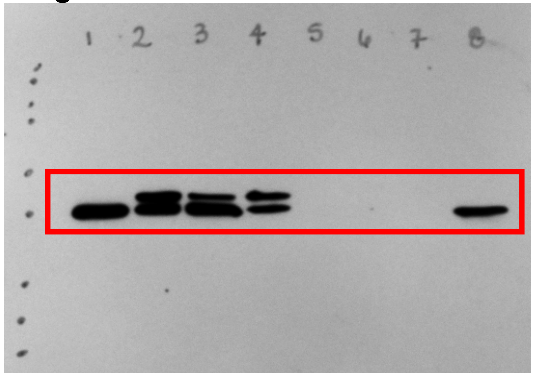

Fig. S9 P-TBK1

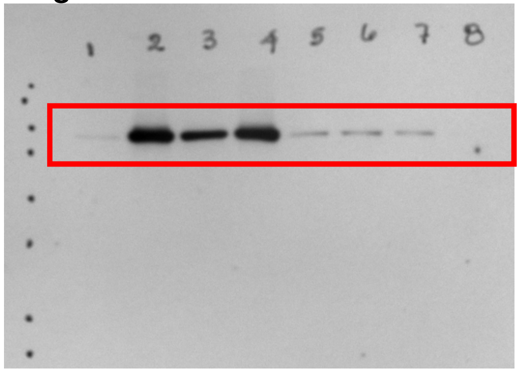

Fig. S9 TBK1

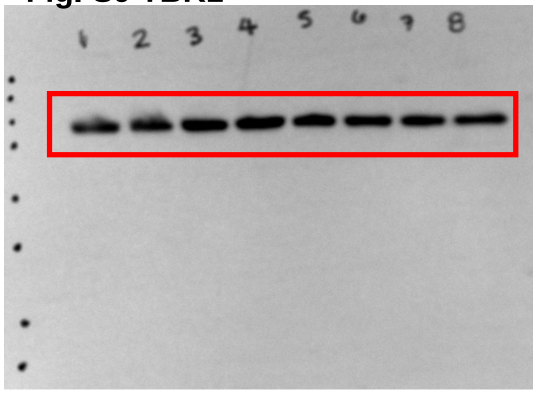

Fig. S9 P-IRF3

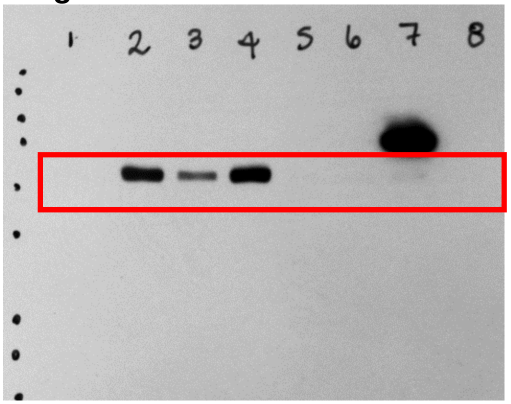

Fig. S9 IRF3

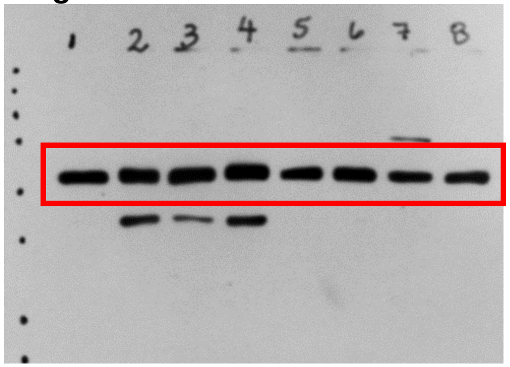

Fig. S9 APOL1

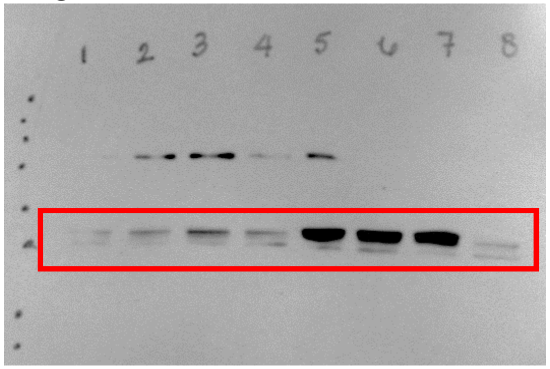

Fig. S9 GAPDH

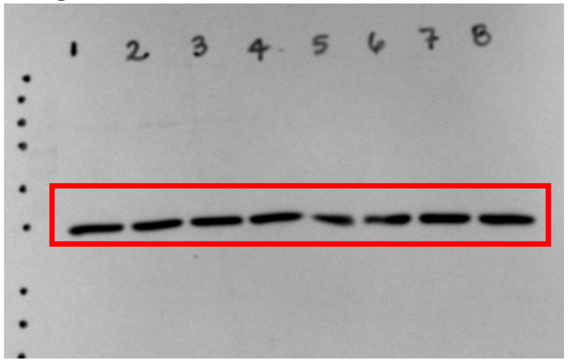

Supplement: Supplementary file 1 — Nucleosomal dsDNA Stimulates APOL1 Expression in Human Cultured Podocytes by Activating the cGAS/IFI16-STING Signaling Pathway [file 41598_2019_51998_MOESM1_ESM.pdf]
